# Mutagenesis of N-terminal residues of feline foamy virus Gag reveals entirely distinct functions during capsid formation, particle assembly, Gag processing and budding

**DOI:** 10.1186/s12977-016-0291-8

**Published:** 2016-08-22

**Authors:** Yang Liu, Matthew J. Betts, Janet Lei, Guochao Wei, Qiuying Bao, Timo Kehl, Robert B. Russell, Martin Löchelt

**Affiliations:** 1 Department of Molecular Diagnostics of Oncogenic Infections, Research Program Infection and Cancer, German Cancer Research Center (DKFZ), Im Neuenheimer Feld 242, 69120 Heidelberg, Germany; 2 CellNetworks, Bioquant, University of Heidelberg, Im Neuenheimer Feld 267, 69120 Heidelberg, Germany; 3 Biochemie Zentrum Heidelberg (BZH), Im Neuenheimer Feld 328, 69120 Heidelberg, Germany; 4 Center for Cancer Research, National Cancer Institute, Frederick, MD USA; 5 Department of Oncology, University of Oxford, Oxford, UK; 6 Biology Department, East China Normal University, Shanghai, China

**Keywords:** Foamy virus, Feline foamy virus, Gag, Mutagenesis, Capsid formation, Assembly, Genome packaging, Particle budding and maturation, Transmission

## Abstract

**Background:**

Foamy viruses (FVs) of the *Spumaretrovirinae* subfamily are distinct retroviruses, with many features of their molecular biology and replication strategy clearly different from those of the Orthoretroviruses, such as human immunodeficiency, murine leukemia, and human T cell lymphotropic viruses. The FV Gag N-terminal region is responsible for capsid formation and particle budding via interaction with Env. However, the critical residues or motifs in this region and their functional interaction are currently ill-defined, especially in non-primate FVs.

**Results:**

Mutagenesis of N-terminal Gag residues of feline FV (FFV) reveals key residues essential for either capsid assembly and/or viral budding via interaction with the FFV Env leader protein (Elp). In an in vitro Gag–Elp interaction screen, Gag mutations abolishing particle assembly also interfered with Elp binding, indicating that Gag assembly is a prerequisite for this highly specific interaction. Gradient sedimentation analyses of cytosolic proteins indicate that wild-type Gag is mostly assembled into virus capsids. Moreover, proteolytic processing of Gag correlates with capsid assembly and is mostly, if not completely, independent from particle budding. In addition, Gag processing correlates with the presence of packaging-competent FFV genomic RNA suggesting that Pol encapsidation via genomic RNA is a prerequisite for Gag processing. Though an appended heterogeneous myristoylation signal rescues Gag particle budding of mutants unable to form capsids or defective in interacting with Elp, it fails to generate infectious particles that co-package Pol, as evidenced by a lack of Gag processing.

**Conclusions:**

Changes in proteolytic Gag processing, intracellular capsid assembly, particle budding and infectivity of defined N-terminal Gag mutants highlight their essential, distinct and only partially overlapping roles during viral assembly and budding. Discussion of these findings will be based on a recent model developed for Gag–Elp interactions in prototype FV.

**Electronic supplementary material:**

The online version of this article (doi:10.1186/s12977-016-0291-8) contains supplementary material, which is available to authorized users.

## Background

Retroviruses use two different major assembly and budding pathways. In most Orthoretroviruses including human immunodeficiency viruses (HIV), fully assembled capsids cannot be detected in the cytoplasm, since assembly of Gag and Gag-Pol take place at the plasma membrane at the site of budding [1, 2]. This type of budding pathway is used by type C retroviruses, such as avian sarcoma leukosis virus, murine leukemia virus, HIV and other lentiviruses [3–5]. In the second pathway, Gag assembles into premature capsids within the cytoplasm, which are then transported to the plasma membrane and acquire an envelope upon budding. Type B/D retroviruses, such as mouse mammary tumor virus and Mason-Pfizer monkey virus, are known to use this budding pathway [6–9].

Foamy viruses (FVs) have many unique features that set them apart from most Orthoretroviruses [10]. While Gag and Env structure, processing and the mechanisms driving particle release are FV-specific, FV capsid assembly resembles, at least in some aspects, that of type B/D retroviruses since FV capsid assembly also occurs at the pericentriolar site, around the microtubule organizing center (MTOC) [7, 11, 12]. Secondly, FV Gag is not N-terminally myristoylated like in most Orthoretroviruses. Thirdly, FV Gag particle budding relies absolutely on the expression of the cognate Env [13, 14]. Thus FV cannot be pseudotyped by foreign glycoproteins, such as VSV-G, since it does not allow the critical Gag-Env interactions needed for release [10]. Finally and unique among retroviruses, Gag in released FV particles is not processed into the mature matrix, capsid and nucleocapsid domains MA, CA, and NC but instead remains largely unprocessed except for a C-terminal cleavage event that is required for particle infectivity [15].

The FV Elp subunit of Env is an abundant and stable component of viral particles, with its N-terminal cytosolic region interacting with the Gag MA layer during budding [13, 14, 16, 17]. Two conserved tryptophan residues were identified as essential for specific interactions between Env and Gag, allowing particle budding [13, 14]. Biophysical analyses of the capsid structure by cryoelectron microscopy and surface plasmon resonance suggest that direct and specific binding between Elp and N-terminal residues of Gag is essential for particle release [13, 14, 16]. Maturation of assembled viral particles requires the activity of Pol, which is expressed independent of Gag via a spiced, sub-genomic transcript [18, 19]. The FV Pol precursor is auto-catalytically processed by the protease (PR) into only two subunits. The larger subunit confers protease/reverse transcriptase/RNase H (PR-RT-RN) enzymatic activities, while the smaller subunit has integrase (IN) activity [15, 20]. Genomic RNA is required for efficient Pol incorporation into viral particles [21, 22].

A number of functional domains or motifs in prototype/primate FV (PFV) Gag have been proposed by bioinformatics or functionally characterized. Four predicted coiled-coil (CC) motifs may exist in PFV Gag. The CC1 motif is located at the extreme N-terminus (residues 4–19). Studies indicated that this region is responsible for interaction with Env [23, 24]. The CC2 motif (133–146) is also located at the N-terminus and may be crucial for Gag–Gag interaction during viral assembly, similar to those mediated in MA domains of the Orthoretroviruses [25]. The CC3 motif (161–174) is mainly involved in interaction between Gag molecules for capsid formation and the light chains of dynein motor protein complexes, which facilitate viral particle transport to the MTOC [26]. The biological function of the CC4 motif, which lies upstream of three glycine-arginine (GR) rich boxes in PFV responsible for genome packaging, nuclear localization and reverse transcription [27–29], has not yet been characterized. In other FVs, a less defined GR-rich region replaces these boxes [30]. Close to the N-terminus of PFV Gag, a cytoplasmic targeting and retention signal (CTRS) homologous to that of the B/D morphotype retroviruses is responsible for directing Gag to the MTOC, the cytoplasmic capsid assembly site [11, 12]. The CTRS domain consists of approximately 16 amino acids centered around a critical arginine (R) at position 50 in PFV Gag [11]. Mutation of this central Arg results in a block in capsid assembly and viral budding, even in the presence of Env [11]. Finally, an efficiently utilized cleavage site of the viral PR lies close to the C-terminus of Gag [15]. Due to the intracellular capsid assembly, budding of FV particles into intracellular membrane compartments and their relatively inefficient release, Gag processing can already be observed in cell-associated Gag and Pol proteins [15].

Phylogenetic analyses indicate that PFV Gag is distantly related to the non-primate FVs, such as feline, bovine, and equine FVs (FFV, BFV and EFV, respectively) [31]. Moreover, many predicted structural domains or motifs in PFV Gag are different compared to the non-primate FVs. For instance, the GR boxes of primate FVs are replaced by less defined arrays of glycine and arginine residues and a 100–130 amino acid insertion is present in PFV and simian FV Gags compared to FFV, EFV and BFV starting at around Gag residue 160 [30]. For a deeper understanding of the function of FV Gag during viral replication, our studies were extended to FFV by focusing on the characterization of the FFV Gag N-terminal residues essential for viral assembly and budding. We identify key determinants harbored within the N-terminus of FFV Gag responsible for capsid assembly, budding and interaction with Env and Elp. We also analyzed the influence of Pol and genomic RNA on Gag processing, particle assembly and release.

## Results

### N-terminal deletions of FFV Gag interfere with particle budding

**Fig. 1:**
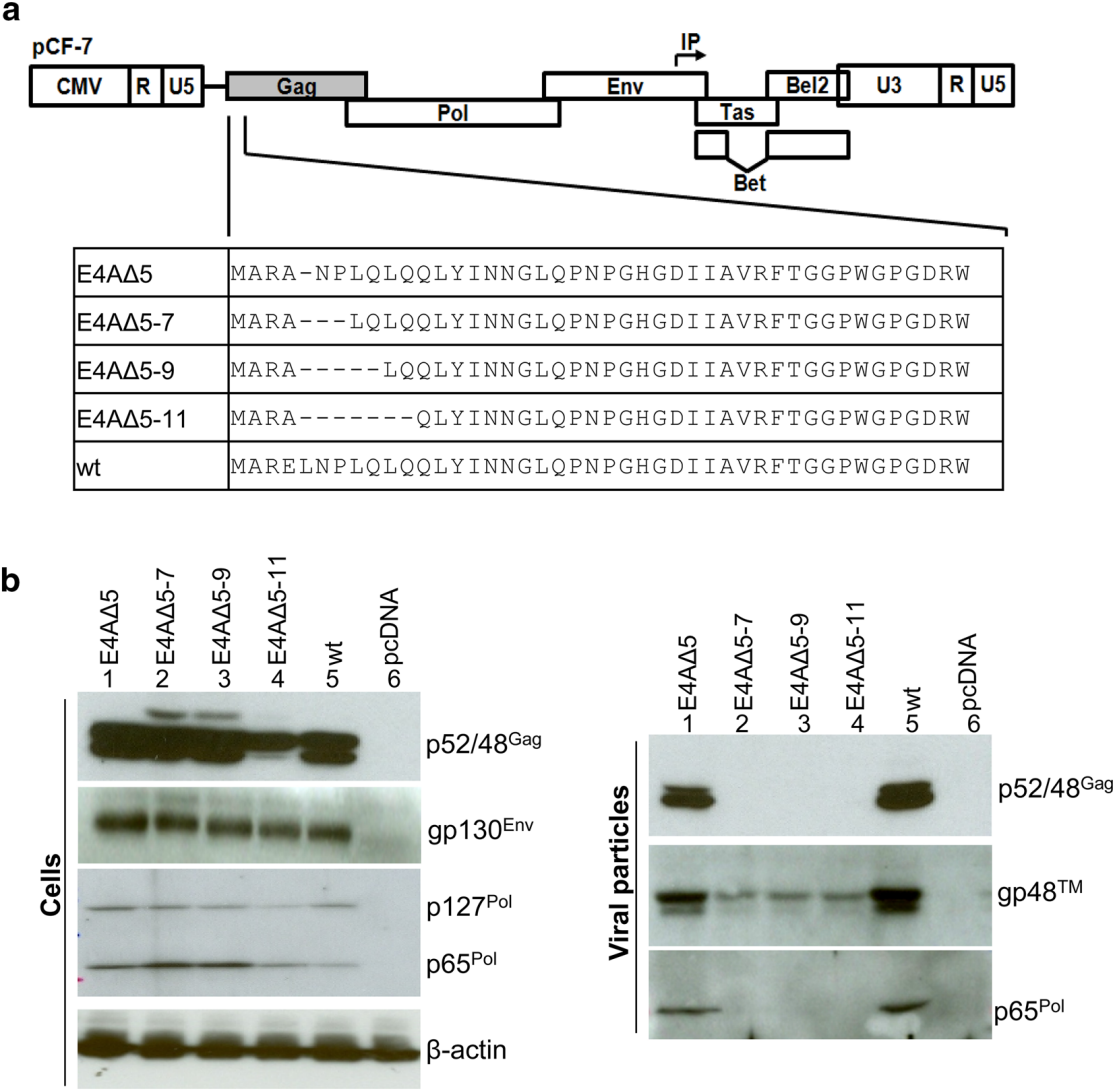
Phenotype of N-terminal Gag deletion mutants. **a** Schematic of the FFV N-terminal Gag deletion mutants. Four Gag mutants (E4AΔ5, E4AΔ7, E4AΔ9, E4AΔ11) were constructed based on the proviral clone pCF-7. **b** 293T cells were transfected in 10 cm dishes with 8 µg of proviral pCF-7-based Gag mutants (*lanes 1–4*), the wild-type parental pCF-7 (*lane 5*) or pcDNA (*lane 6*). Aliquots of cell lysates and VLPs in supernatants were analyzed by SDS-PAGE. Positions of Gag p48 and p52, full-length Env precursor gp130^Env^, mature processed Env gp48™, Pol precursor p127^Pol^ and PR-RT-RN domain p65^Pol^ are marked

To analyze whether the N terminus of FFV Gag is responsible for particle budding, increasing amino acid deletions together with a cloning-associated Gag-E4A exchange [32], were introduced into *gag* in the wild-type (wt) proviral clone pCF-7 (Fig. 1a). 293T cells transfected with mutant and wt FFV genomes were analyzed 2 days post-transfection (p.t.). Mutant and wt Gag proteins were expressed at comparable levels in the cells. However, FV-specific Gag processing at the C-terminus was impaired in E4A∆5-11. Expression of the Env gp130^Env^ precursor was comparable for all clones, while levels of the Pol precursor and the PR-RT-RNaseH domain (p127^Pol^ and p65^Pol^, respectively) varied among the clones (Fig. 1b, left panel). Cleared culture supernatants were sedimented through 20 % sucrose to detect particle release. The budding capacity of E4A∆5 Gag particles containing gp48™ (TM) and p65^Pol^ was similar to that of wt Gag (Fig. 1b, right panel, lanes 1 and 5). However, deletion of two or more amino acids displayed low-level Env-only subviral particle budding (Fig. 1b, left panel, lanes 2–4) [33, 34]. Release of Gag- and Pol-containing virions or VLPs was undetectable. In line with these data, only mutant E4A∆5 maintained wt infectivity; all other mutants were non-infectious (data not shown). Taken together, these data substantiate the hypothesis that the integrity of N-terminal Gag sequences is required for full biological activity of Gag and the assembly and release of wt FV particles.

### N-terminal Gag motifs essential for FFV particle release and infectivity

**Fig. 2:**
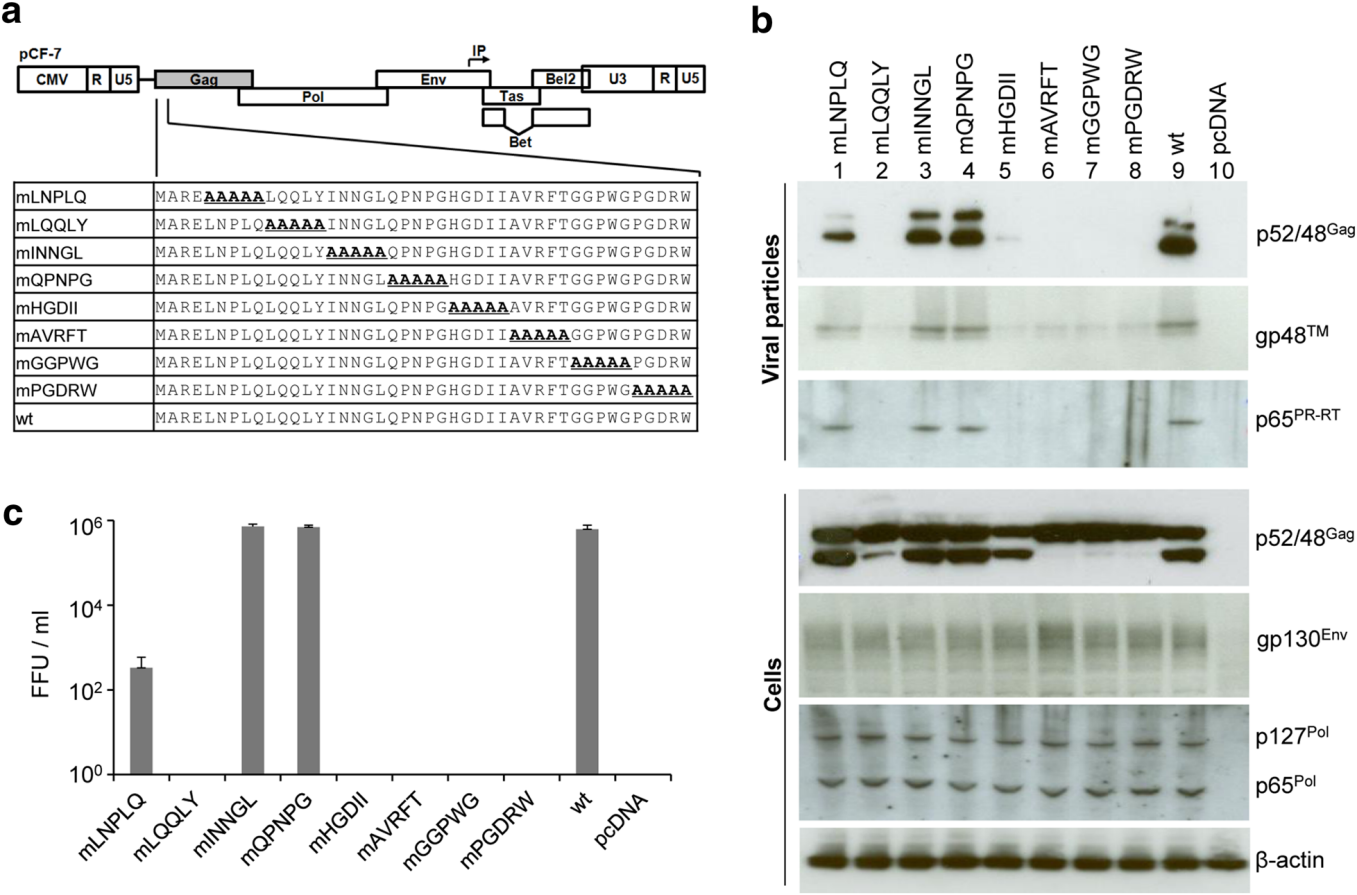
Budding of defined FFV five-alanine Gag mutants. **a** Five-alanine scanning mutagenesis at the N-terminus of FFV Gag. Eight Gag mutants (mLNPLQ, mLQQLY, mINNGL, mQPNPG, mHGDII, mAVRFT, mGGPWG and mPGDRW) were constructed based on the proviral clone pCF-7 or the Gag expression plasmid pBC-Gag-oPRE. m indicates a replacement of the original sequence with five alanines (*underlined*). **b** 293T cells were transfected in 10 cm dishes with 8 µg of each proviral pCF-7-based Gag mutant (mLNPLQ, mLQQLY, mINNGL, mQPNPG, mHGDII, mAVRFT, mGGPWG or mPGDRW) (*lanes 1–8*), pCF-7 (*lane 9*) or pcDNA (*lane 10*). Aliquots of cell lysates and VLPs in supernatants were analyzed by SDS-PAGE. Positions of Gag p48 and p52, full-length Env precursor gp130^Env^; mature processed Env gp48™, Pol precursor p127^Pol^ and PR-RT-RN domain p65^Pol^ are marked. **c** Two days p.t., released particles were titrated onto FeFab cells. Mean titers of three independent experiments are given. *Error bars* represent the standard deviation

To identify Gag motifs essential for viral particle formation and budding, FFV Gag residues 5-44 in pCF-7 were mutated in blocks of five by alanine scanning mutagenesis (Fig. 2a). As above, protein expression in transfected cells, infectivity and particle release were analyzed 2 days p.t. Intracellular expression of FFV Env and Pol (Fig. 2b, lower panels) was not affected. However, C-terminal processing of Gag was abrogated (clones mAVRFT, mGGPWG and mPGDRW) or strongly impaired (clones mLQQLY and mHGDII). Gag processing in mutants mLNPLQ, mINNGL and mQPNPG was similar to that of wt.

The budding efficacy of the proviral mutants mINNGL and mQPNPG, as determined by Gag, Pol and Env detection in particulate supernatants after sucrose cushion centrifugation, was similar to the wt (Fig. 2b, upper panels). Clone mLNPLQ displayed reproducibly lower levels of particle release. Particle release by Gag mutants mLQQLY, mHGDII, mAVRFT, mGGPWD and mPGDRW was undetectable in culture supernatants (Fig. 2b, lanes 2 and 5–8). Budding of mutants was fully reflected by their infectivity, as measured by titration on FeFab cells. Budding-competent mutants mINNGL and mQPNPG retained wt infectivity, while mLNPLQ with impaired budding showed a more than 10^3^-fold reduction in infectivity (Fig. 2c). Budding-incompetent Gag mutants mLQQLY, mHGDII, mAVRFT, mGGPWG and mPGDRW were completely non-infectious. Additionally, cells expressing proviral Gag mutants mLQQLY, mHGDII, mAVRFT, mGGPWG and mPGDRW did not exhibit cell fusion activity whereas cells expressing mLNPLQ, mINNGL, mQPNPG and wt FFV Gag proteins displayed the characteristic syncytia indicated by large and GFP-positive cell fusions that are a hallmark for wt FFV-infected cells (Additional file 1: Figure S1A).

The different budding, proteolytic Gag processing, syncytia formation, and infectivity phenotypes of these N-terminal proviral Gag mutants imply multiple functions for N-terminal residues during capsid assembly and maturation, Env interaction and particle release. In 293T cells, syncytia formation is greatly enhanced upon co-expression of wt Gag, possibly due to Gag-mediated surface targeting or capsid-induced Env clustering (YL and ML, unpublished observations). Furthermore, while mutants mLQQLY and mHGDII are defective in syncytia formation, particle release and infectivity, mutants mAVRFT, mGGPWG and mPGDRW are additionally defective in intracellular Gag processing. Based on these observations and published data and concepts for FV cytosolic capsid assembly, Gag processing and Env-dependent particle release [9], it is likely that Gag assembly is completely abrogated in mutants mAVRFT, mGGPWG and mPGDRW as indicated by the absence of Gag processing that is assumed to be dependent on capsid assembly. Since at least a certain degree of Gag processing occurs in mutants mLQQLY and mHGDII, capsid assembly and Gag processing may be still functional and these mutants are only deficient in particle release. The rest of the mutants displayed a wt phenotype or only a clearly reduced viral infectivity (mLNPLQ).

### Mutations of Gag residues 30–44 disrupt particle assembly

**Fig. 3:**
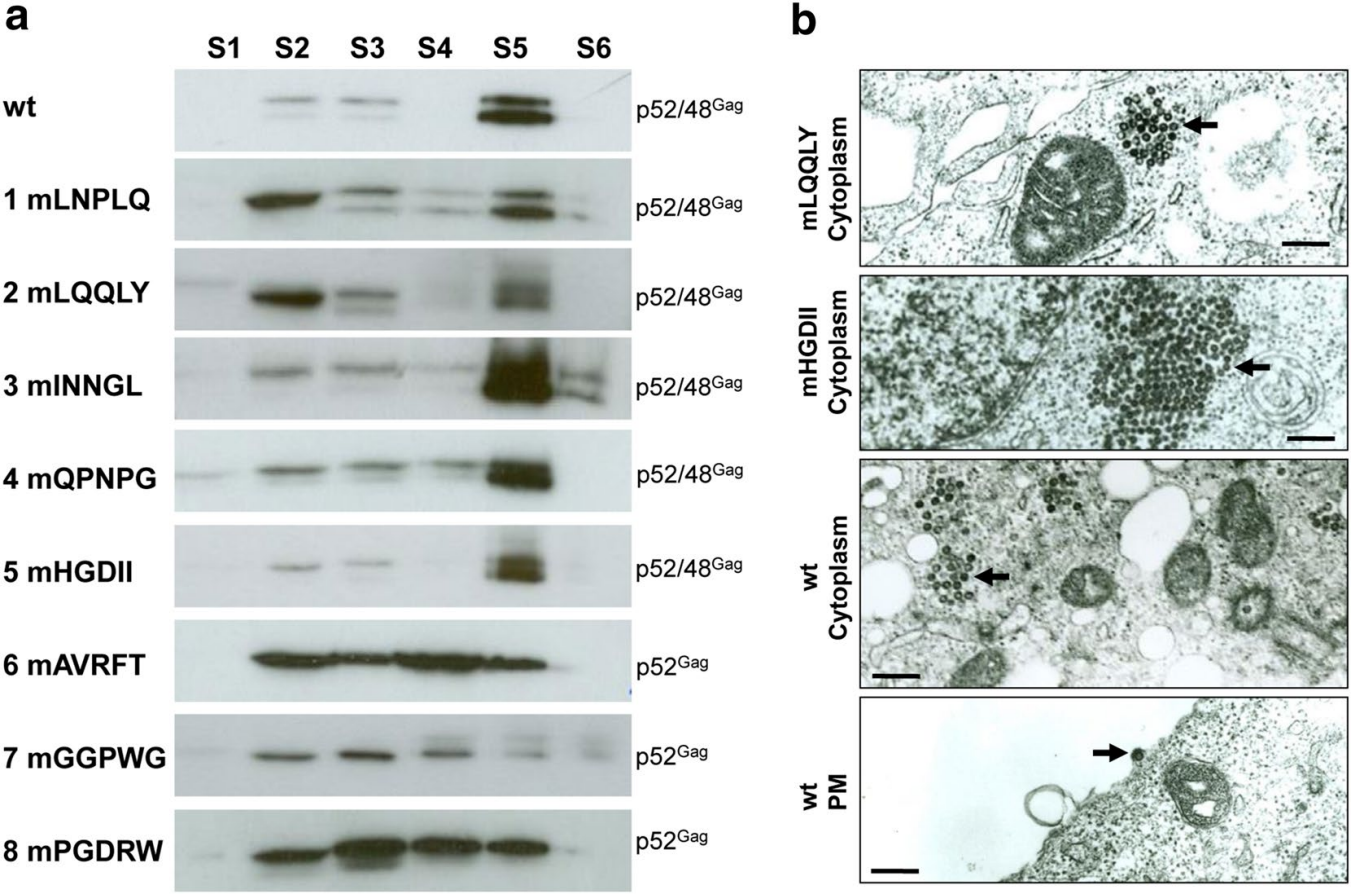
Intracellular capsid assembly of FFV Gag mutants determined by sedimentation and transmission electron microscopy analyses. **a** 293T cells were transfected in 10 cm dishes with 8 µg of proviral pCF-7-based Gag mutants (mLNPLQ, mLQQLY, mINNGL, mQPNPG, mHGDII, mAVRFT, mGGPWG or mPGDRW) or the parental provirus pCF-7. Two days p.t., cytoplasmic extracts were prepared and used for sucrose gradient sedimentation. Each six fractions (*S1*–*S6*) were collected from the top of the gradient and analyzed by immunoblotting. Positions of FFV Gag p48 and p52 are marked. **b** Intracellular capsid assembly of wt Gag and Gag mutants mLQQLY and mHGDII were visualized by transmission electron microscopy of transfected and fixed 293T cells. Transmission electron micrographs show representative thin sections of 293T cells transiently transfected with pCF-7-based plasmids expressing mLQQLY, mHGDII or wt Gag. Capsids are marked by *black arrows*. *Scale bars* are 500 nm in length

To study capsid assembly of wt and mutant Gag proteins, the corresponding proviruses were transfected into 293T cells. Cytosolic fractions were harvested 2 days p.t. using a method established for PFV but employing a tenfold reduced concentration of a non-ionic detergent [11]. Sucrose gradient sedimentation was subsequently used to determine whether mutant Gag proteins have the capacity to assemble into cytosolic FV capsids [11]. wt FFV capsids accumulated mainly in fraction 5 (Fig. 3a, top panel, fraction S5). The absence of Gag proteins in the top fraction 1 (S1) suggests that most, if not all, cytosolic Gag proteins entered the gradient as higher molecular forms; free, non-assembled wt Gag was rare. Faint bands in fraction 2 and 3 (S2, S3) were interpreted as sub-capsid Gag assemblies, such as capsomeres with lower sedimentation coefficients corresponding to their presence in 10–30 % sucrose.

The infectious and budding-competent proviral Gag mutants mLNPLQ, mINNGL and mQPNPG and budding-incompetent mutants mLQQLY and mHGDII, which retained Gag processing, did not show any obvious phenotypic changes in Gag distribution compared to wt Gag. As expected, high amounts of full-length and processed Gag were detected in the more dense capsid fraction S5. Low to moderate amounts of less defined Gag assemblies were detected in fractions S2–S4. In stark contrast, the more distal Gag mutants mAVRFT, mGGPWG and mPGDRW, with mutations between Gag residues 30–44, exhibited unprocessed Gag proteins throughout fractions S2–S4/S5, indicating unspecific Gag aggregation rather than a major and well-defined capsid assembly product. In line with studies on the assembly-relevant R50 residue in the PFV CTRS [11], alanine scanning mutagenesis of the FFV counterpart Gag R43 and its flanking residues (mPGDRW) inhibited FFV capsid assembly.

Intracellular capsid assembly of the wt FFV provirus and mutants mLQQLY, mHGDII and mAVRTF was examined by TEM of correspondingly transfected 293T cells. In wt FFV-transfected cells, intracellularly assembled capsids were mainly detected at the MTOC, with particle budding and release events at the plasma membrane (Fig. 3b, lower panels). Mutants mLQQLY and mHGDII formed capsid structures in the cytoplasm (Fig. 3b) but no particle released events were observed, as expected because of their budding-deficient phenotype (Fig. 2b). The size and morphology of the capsids were indistinguishable from wt FFV, suggesting that these mutations did not alter the gross viral structure. In cells transfected with mutant mAVRFT, assembled capsids were not detectable (data not shown).

### Mapping of N-terminal FFV Gag residues critical for capsid assembly, budding and infectivity

**Fig. 4:**
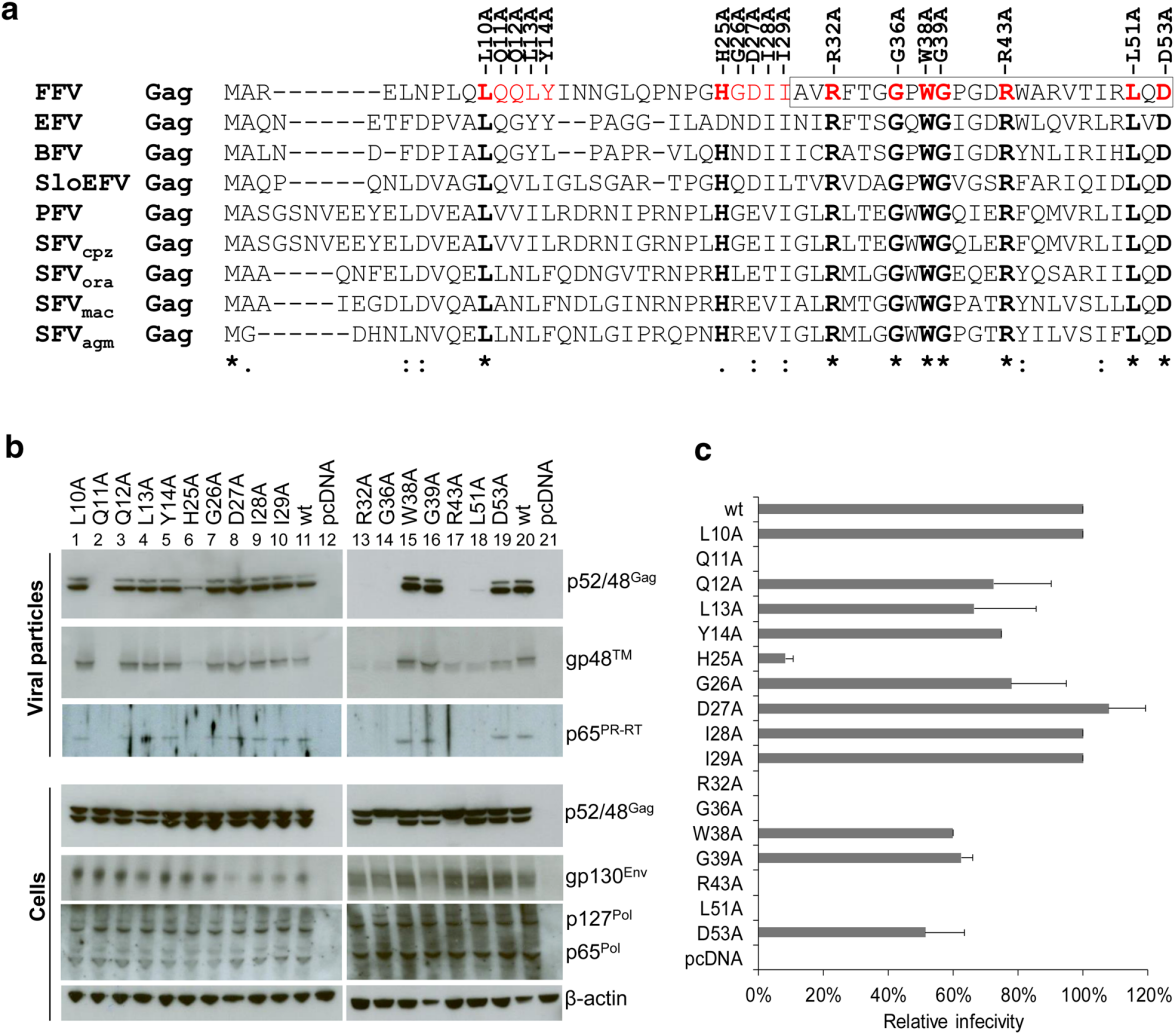
Alanine point mutations of conserved N-terminal Gag residues abolish particle budding, infectivity and Gag processing. **a** Alignment of FV Gag sequences using ClustalW2 Multiple Sequence Alignment and identification of highly conserved residues. Sequences were obtained from SwissProt (FFV Gag, accession number O56860; EFV Gag, accession number Q9J4C8; BFV Gag, accession number O41893; SloEFV Gag from endogenous sloth FV [56]; PFV Gag, accession number P14349; SFVcpz Gag from chimpanzee, accession number Q87039; SFVora from orangutan, accession number P27400; SFVmac Gag from macaque, accession number Q00071; SFVagm from African green monkey, accession number M74895). Highly conserved residues are marked in *bold face letters* and *asterisks* below the alignment. The proposed FFV Gag cytoplasmic targeting and retention signal (CTRS) is marked by a frame. Alanine point mutations of FFV Gag (L10A, Q11A, Q12A, L13A, Y14A, H25A, G26A, D27A, I28A, I29A, R32A, G36A, W38A, G39A, R43A, L51A and D53A, in *red*) were constructed using the proviral vector pCF-7 and the Gag expression clone pBC-Gag-oRPE. **b** 293T cells were transfected in 10 cm dishes with 8 µg of plasmid encoding for proviral Gag mutants (L10A, Q11A, Q12A, L13A, Y14A, H25A, G26A, D27A, I28A, I29A, R32A, G36A, W38A, G39A, R43A, L51A and D53A, *lanes 1–10* and *13–19*, respectively), pCF-7 (*lanes 11* and* 20*) or pcDNA (*lanes 12* and* 21*). Cell lysates and VLPs in supernatants were analyzed by SDS-PAGE. Positions of Gag p48 and p52, full-length Env precursor gp130^Env^, mature processed Env gp48™, Pol precursor p127^Pol^ and PR-RT-RN domain p65^Pol^ are marked.** c** Two days p.t., infectivity was assessed by titration onto FeFab cells. Mean relative titers of three independent titration experiments are shown. *Error bars* represent the standard deviation

Based on the alanine scanning mutagenesis of FFV Gag mutants mLQQLY, mHGDII, mAVRFT, mGGPWG and mPGDRW (Figs. 2, 3), the presence of conserved residues in the N-terminus of FV Gag (Fig. 4a) and published data for PFV [9], individual amino acid replacements in Gag were engineered, generating single amino acid proviral Gag mutants pCF-7-L10A, Q11A, Q12A, L13A, Y14A, H25A, G26A, D27A, I28A, I29A, R32A, G36A, W38A, G39A, R43A, L51A and D53A (Fig. 4a).

In 293T cells transfected with these single amino acid substitution mutants, similar amounts of wt and mutated Gag, Env and Pol proteins were detected (Fig. 4b, bottom panel), suggesting that the mutations did not affect overall protein abundance. Importantly, Gag processing was absent in mutants G36A and R43A. In addition, the Gag mutants had different budding capacities. Mutants L10A, Q12A, L13A, Y14A, G26A, D27A, I28A, I29A, W38A, G39A and D53A exhibited wt budding with processed Gag, Pol and Env in the particle fraction (Fig. 4b, top panels). In contrast, mutant H25A had significantly impaired budding and Q11A, R32A, G36A, R43A and L51A did not bud at all (Fig. 4b, top panels). Viral titers of all budding-competent mutants were within a two-fold range of the wt, whereas the low-level budding mutant H25A retained only 10 % of wt infectivity (Fig. 4c). All infectious mutants induced syncytia in transfected cells (Additional file 1: Figure S1B). H25A partially lost cell fusion activity, as shown by the smaller syncytia size while Q11A, R32A, G36A, R43A and L51A also did not lead to any syncytia formation (Additional file 1: Figure S1B).

As anticipated, budding-deficient mutants (Q11A, R32A, G36A, R43A, and L51A) were not infectious (Fig. 4b, c). Strikingly, the budding-incompetent proviral Gag mutants Q11A, R32A and L51A displayed the double bands indicative of Gag processing, while G36A and R43A completely lost proteolytic Gag processing (Fig. 4b, lanes 2, 13, 14, 17, and 18). The phenotype of alanine scanning mutant ∆HGDII is partly reflected by the H25A mutant. In contrast, mAVRFT displayed a complete loss of Gag functions (capsid formation, processing and budding), while mutagenesis of only the central arginine (R32) impaired particle release but not Gag processing and capsid assembly (see below). The distinct phenotypes of these N-terminal Gag mutants with respect to budding capacity, proteolytic processing and infectivity imply different roles for individual residues during assembly and budding.

### Most Gag point mutations show intact capsid formation

**Fig. 5:**
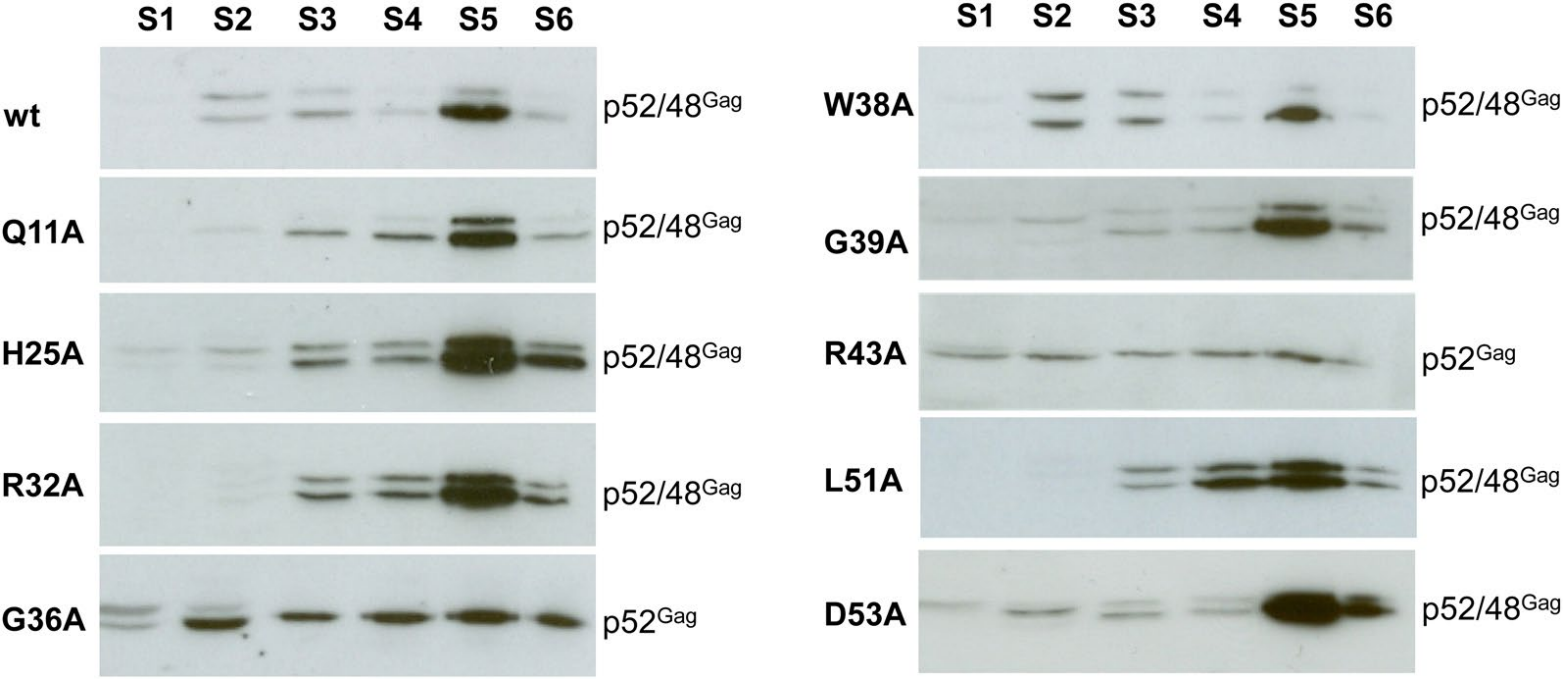
Gradient analysis of intracellular mutant Gag capsids. 293T cells were transfected in 10 cm dishes with 8 µg of pCF-7-Q11A, H25A, R32A, G36A, W38A, G39A, R43A, L51A, D53A or wild-type pCF-7. Two days p.t., cytoplasmic extracts of transfected 293T cells were prepared and used for sucrose gradient sedimentation. Each, six fractions (S1–S6, 700 μl) were collected from the top of the gradient and analyzed by immunoblotting. Positions of FFV Gag p48 and p52 are marked

Capsid assembly of budding-incompetent mutants Q11A, H25A, R32A, G36A, R43A, and L51A was compared to mutant D53A and pCF-7 by sucrose gradient analyses of cytosolic extracts. The distribution of processed and unprocessed Gag proteins of Q11A, H25A, R32A, G39A, and L51A was similar to that of the wt and wt-like D53A, with capsids sedimenting in fraction S5. In contrast, mutant G36A and R43A Gag proteins were evenly distributed throughout the gradients as a single unprocessed form of Gag, likely representing proteins aggregates and a lack of proper capsid assembly (Fig. 5). This clearly reflects the phenotype of the parental alanine scanning mutants mGGPWG and mPGDRW (Fig. 3a). The phenotype of FFV Gag R43A agrees with its corresponding mutation in PFV Gag (R50) [11]. Importantly, the alanine scanning mutants and single amino acid replacements show a clear correlation of capsid assembly and proteolytic Gag processing, indicating that capsid assembly is a prerequisite for, among other things, Pol incorporation into the viral particle and PR activation. Thus, lack of capsid formation by mutants G36A and R43A may be responsible for downstream events, highlighting the complexity and interdependency of individual assembly steps.

### Assembly-incompetent mutant Gag proteins localize to the nucleus

**Fig. 6:**
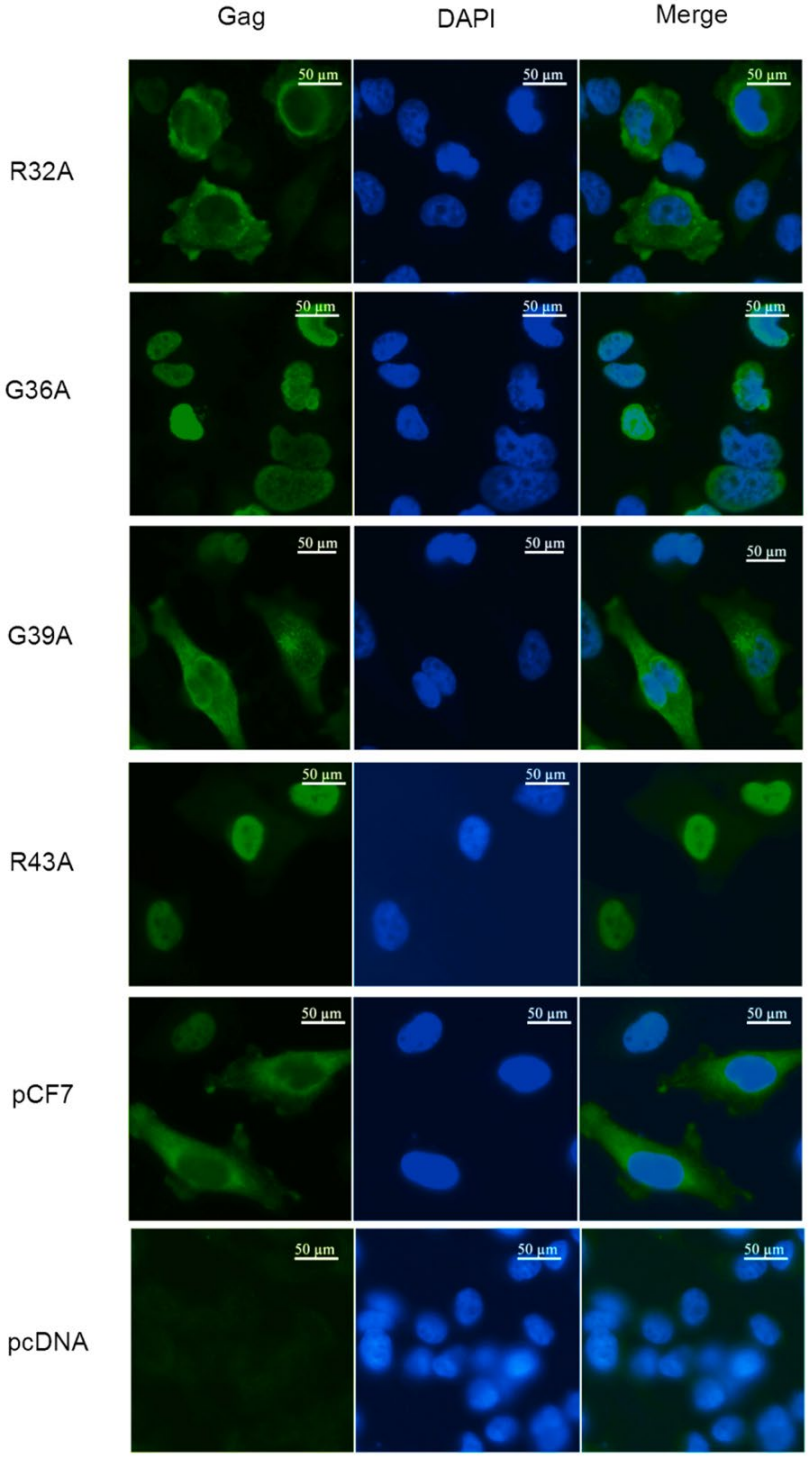
Subcellular localization of wt and single-mutant FFV Gag proteins. HeLa cells were transfected with proviral pCF-7-based Gag mutants (R32A, G36A, G39A and R43A) and the parental provirus pCF-7. pcDNA plasmid was used for transfection as negative control. At 36 h p.t., cells were fixed and stained with a rabbit polyclonal antiserum generated against FFV Gag and Alexa-488-conjugated secondary antibody. Nuclei were stained with DAPI. *Scale bars* represent 50 µm

We next studied the subcellular localization of the different FFV Gag mutants in the proviral context. Transfection with all five alanine scanning mutants and controls was conducted using HeLa cells since they allow better visualization of subcellular structures compared to 293T cells which have only a very narrow rim of cytosol around the nucleus. Cells were fixed 36 h p.t. using paraformaldehyde and processed for FFV Gag IFF using a MA-specific polyclonal antiserum. While all capsid assembly-competent mutant Gag proteins showed mostly cytosolic and perinuclear subcellular localization similar to wt Gag, the assembly-deficient mutants mHGDII, mAVRFT, mGGPWG and mPGDRW were mainly or exclusively localized to the nucleus (Additional file 2: Figure S2). To refine these analyses, the single amino acid FFV Gag mutants Q11A, H25A, R32A, G36A, W38A, G39A, R43A, L51A, D53A, the wt provirus pCF7 and empty vector pcDNA were similarly analyzed, some of these IIFs are shown in Fig. 6. Overall, only FFV Gag mutants G36A and R43A displayed prominent nuclear localization of Gag (above 90 % of cells with nuclear Gag, with the remainder either cytosolic only or nuclear and cytosolic). All other capsid assembly-competent mutants, independent of their budding capacity or infectivity, were concentrated in the cytosol and around the nucleus, similar to wt Gag (56–75 % of cells with cytosolic Gag, 0–14 % nuclear and 10–43 % in both compartments; Fig. 6 and data not shown). Importantly, none of the Gag mutants displayed gross protein aggregation.

### Defined point mutations in Gag abolish the Gag–Elp interaction

Point mutations of conserved key residues in the N-terminus of FFV Gag inhibit viral egress at different stages of viral replication, such as CTRS residues G36A and R43A, which block Gag assembly and processing and thereby particle budding. On the other hand, mutants R32A and L51A form intracellular capsids but are completely incapable of budding and are non-infectious. One plausible explanation for the latter phenotype is that these mutations interrupt the interaction of preformed capsids with Elp essential for membrane targeting and budding out of the cell.

**Fig. 7:**
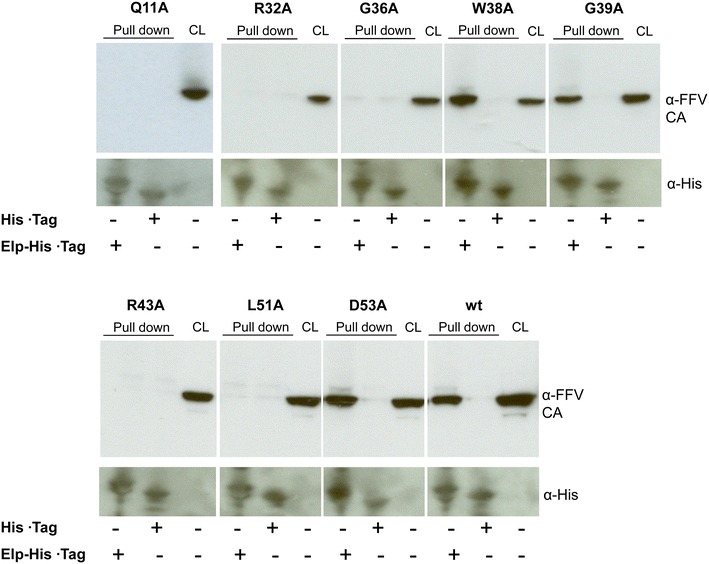
Mutations of specific residues in FFV Gag abolish its interaction with Elp. Cell lysates (CL) were prepared by transfecting 293T cells in 10 cm dishes with 8 µg of plasmid encoding Gag mutant (R32A, G36A, W38A, G39A, R43A, L51A or D53A) or wt Gag and used for protein pull down studies employing the recombinantly expressed cytosolic domain of Elp. Cells transfected with a plasmid encoding for wt Gag or an empty His-tag vector were used as positive or negative controls, respectively. The positions of FFV Gag p52 and His-tagged Elp are marked

The interaction of Elp and Gag in the budding-incompetent mutants was further investigated by pull-down assays using provirus-transfected 293T cells. For this purpose, 293T cells were transfected with plasmids encoding the wt (pCF-7) and proviral Gag mutants Q11A, R32A, G36A, W38A, G39A, R43A, L51A and D53A. Cell lysates (CL) were incubated with the bacterially expressed and purified cytosolic domain of Elp containing the conserved Gag interaction domain [13, 14]. The budding-incompetent mutants Q11A, R32A, G36A, R43A and L51A (regardless of capsid formation capacity) were not precipitated by His-tagged Elp (Fig. 7), indicating that the Gag–Elp interaction was abolished and only assembled capsids are bound by Elp. An empty His-tag plasmid was used as negative control to precipitate Gag-expressing 293T cell lysates, providing a non-relevant background binding in all cases. As expected, wt and wt-like mutants W38A, G39A, D53A bound strongly to His-tagged Elp and were pulled down from the cell lysates.

### FFV capsid formation and Pol and genome encapsidation are required for Gag processing

**Fig. 8:**
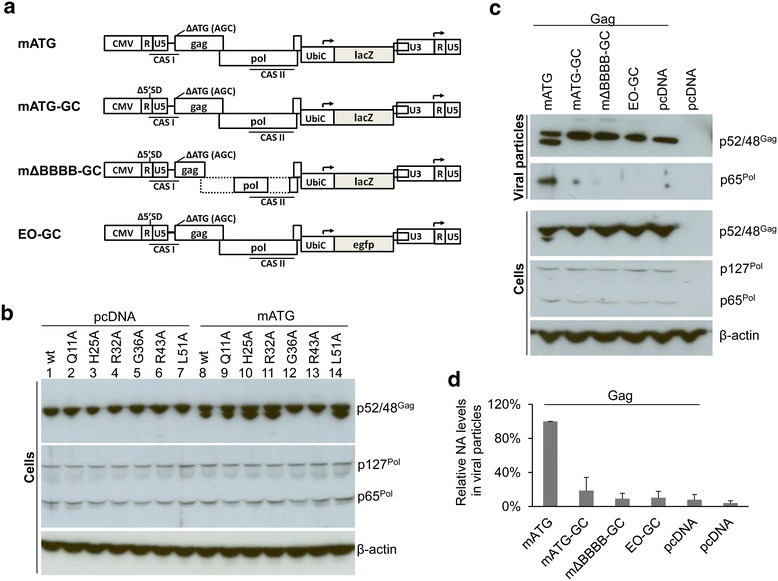
Gag processing requires both Pol and the viral genome. **a** Schematic structures of the four FFV genomes mATG, mATG-GC, mΔBBBB-GC and EO-GC. **b** 293T cells were co-transfected in 10 cm dishes with 2 µg of pBC-Gag-oPRE-based Gag constructs (wt, Q11A, H25A, R32A, G36A, R43A or L51A), 2 µg pMP-Pol-oPRE and 2 µg of the genome mATG. The viral genome was substituted by pcDNA as a negative control. Cell lysates were analyzed by SDS-PAGE. Positions of Gag p48 and p52, Pol precursor p127^Pol^ and PR-RT-RN domain p65^Pol^ are marked. **c** 293T cells were co-transfected in 10 cm dishes with 2 µg pBC-Gag-oPRE, 2 µg pMP-Pol-oPRE, 2 µg pBC-Env and 2 µg of each of four vector genomes. pcDNA was used as the negative control. Cell lysates and VLPs in supernatants were analyzed by SDS-PAGE. Positions of Gag p48 and p52, Pol precursor p127^Pol^ and PR-RT-RN domain p65^Pol^ are marked. **d** Viral RNAs in Gag particles were quantified by qRT-PCR. The figure shows an average of two independent experiments, with *error bars* indicating the range. Data is given as the relative number of cDNA copies compared to the RNA packaging-competent vector mATG, which was set to a value of 100 %

The previous results indicate that Gag processing is tightly linked to capsid formation. We thus determined whether capsid formation and concomitant encapsidation of the RNA genome together with the bound Pol protein is a prerequisite for Gag processing [21]. 293T cells were co-transfected with subgenomic wt and mutant Gag Q11A, H25A, R32A, G36A, R43A and L51A expression vectors, the Pol expression vector pMP-Pol-oPRE and the Gag-deficient but packaging-competent FFV genome mATG. pcDNA was co-transfected instead of mATG to serve as a negative control (Fig. 8a) [35]. Although the vector genome may also direct Pol expression, both, the processed (p65^Pol^) and unprocessed Pol precursor (p127^Pol^) proteins were detected at similar levels in all samples (Fig. 8b). While wt and mutant Gag did not undergo PR processing in the absence of the FFV vector genome (Fig. 8b, lanes 1–7), capsid assembly-competent Gag mutants Q11A, H25A, R32A and L51A processed Gag at a level similar to the wt in the presence of genomic RNA. As anticipated, only assembly-deficient Gag mutant proteins G36A and R43A remained completely unprocessed (Fig. 8b, lanes 8–14).

To further examine the role of viral RNA in Pol encapsidation and subsequent PR activity, we used a subviral system with RNA packaging-deficient mutant vector genomes as additional controls (Fig. 8a). 293T cells were co-transfected with pBC-Gag-oPRE, pMP-Pol-oPRE and pBC-Env encoding wt Gag, Pol and Env, together with pcDNA serving as the negative control or the packaging-competent vector genome mATG or packaging-deficient genomes mATG-GC, mΔBBBB-GC and EO-GC. As shown in Fig. 8a, in all vectors the *env*-*bel* gene region of FFV is replaced by an Ubi-LacZ or Ubi-egfp reporter gene cassette. In mATG, only Gag expression is abrogated by Gag start codon mutagenesis while Pol can be still expressed. In contrast, mutagenesis of the major 5′ splice donor (suffix GC) abrogates genome packaging in mATG-GC. In the genomes mΔBBBB-GC, essential packaging sequences in *pol* are deleted while vector EO-GC carries an egfp instead of the lacZ reporter gene in a background similar to mATG-GC [35, 36]. Regular aliquots of cell lysates and VLPs were purified and analyzed by immunoblotting. Protein expression and particle release, as measured by detection of Gag in each fraction, were comparable for all co-transfections (Fig. 8c). Compared to the RNA packaging-competent vector mATG (Fig. 8c, lane 1) inducing release of particle containing processed Gag and Pol, the packaging-deficient vector mATG-GC, mΔBBBB-GC and EO-GC and control plasmid pcDNA (Fig. 8c, lanes 2–5, respectively) resulted in the complete loss of Pol encapsidation and Gag processing in released VLPs. As particle release was comparable for all co-transfections (Fig. 8c, lanes 1–5), we determined the RNA content in these released particles by qRT-PCR for the ubiquitin C promoter, contained in all vector genomes. A sharp decrease of the RNA content in the RNA packaging-deficient vectors (below 20 %) compared to that of the RNA packaging-competent vector was observed (Fig. 8d). Together, these data indicate that a packaging-competent genome is not required for capsid formation and that Pol expression alone is insufficient for Gag processing unless Pol is packaged into viral capsids via the viral genome containing the Pol encapsidation signal [21].

### Myr signal rescues capsid assembly and release but abolishes Gag processing and Pol encapsidation

**Fig. 9:**
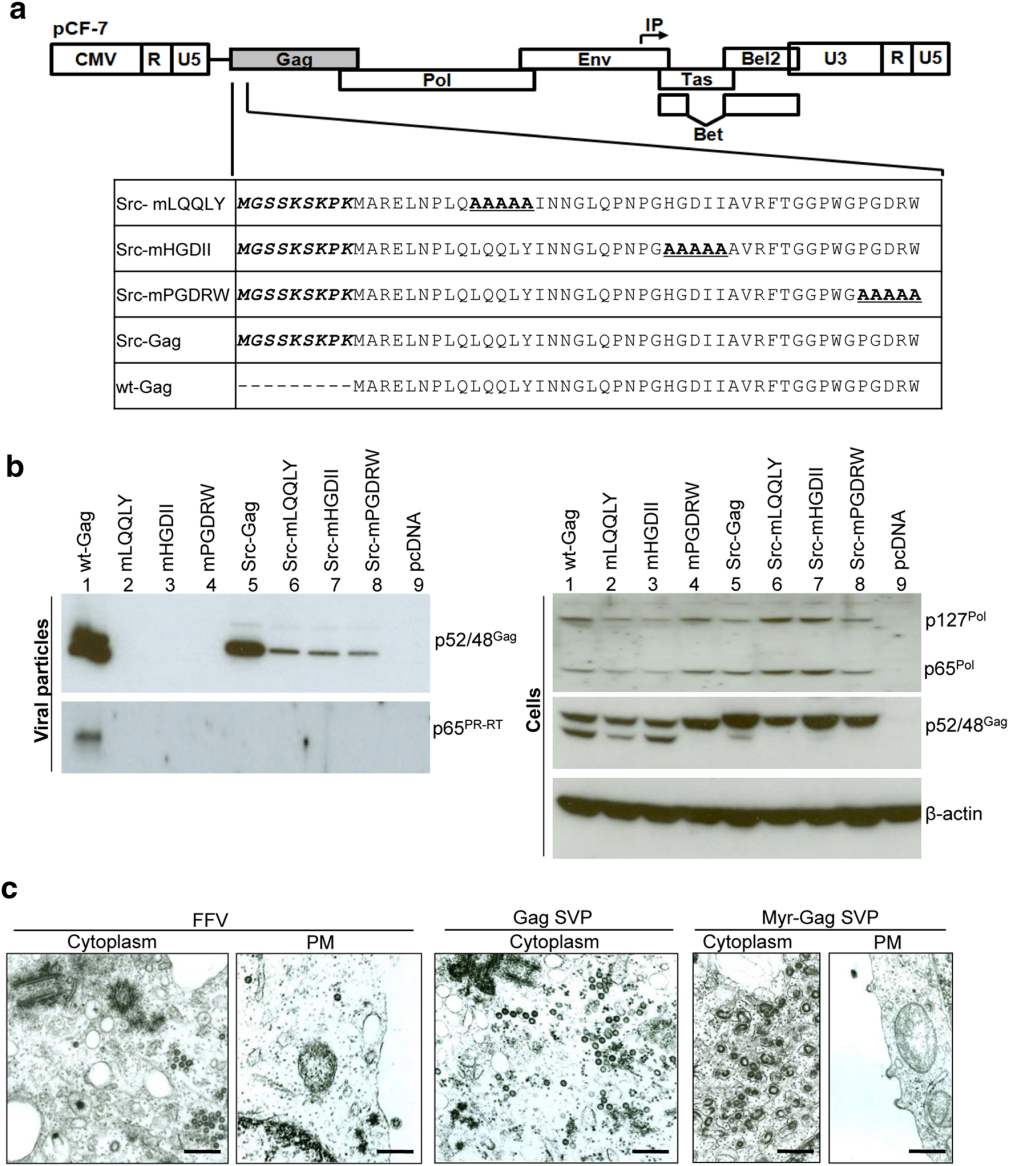
Pol is not incorporated into myr-Gag capsids. **a** Construction of FFV proviral Src-Gag mutants in the pCF-7 provirus. A myr signal derived from the human cellular protein Src was added onto the N-termini of FFV wt Gag and Gag mutants mLQQLY, mHGDII and mPGDRW (shown in *Italic* and *bold*). **b** 293T cells were transfected in 10 cm dishes with 8 µg of proviral pCF-7-based wt Gag (*lane 1*), Gag mutants (mLQQLY, mHGDII, mPGDRW, Src-Gag, Src-mLQQLY, Src-mHGDII and Src-mPGDRW) (*lanes 2–8*), or pcDNA (*lane 10*). Cell lysates and VLPs in supernatants were analyzed by SDS-PAGE. Positions of Gag p48 and p52, Pol precursor p127^Pol^ and PR-RT-RN domain p65^Pol^ are marked. **c** Intracellular capsid assembly of FFV virus, Gag sub-viral particles (SVPs) and Src-Gag SVPs were visualized by transmission electron microscopy of transfected and fixed 293T cells. Transmission electron micrographs show representative thin sections of the cells. *Scale bars* are 500 nm in length

To investigate the determinants essential for Gag processing upon capsid assembly, we engineered FFV wt and Gag mutants mLQQLY (budding-deficient), mHGDII (budding-compromised) and mPGDRW (capsid assembly and Gag processing-deficient) with a heterologous myr motif (src), known to rescue capsid assembly by retargeting Gag from the MTOC to the plasma membrane and generating extracellular but non-infectious viral particles (Fig. 9a) [23, 32, 37]. In all tested myr-Gag genomes, particle budding was partially restored, indicative of capsid assembly (Fig. 9b). However, Pol-mediated processing of wt and mutant myristoylated Gag was completely or dramatically (Src-Gag) reduced in cellular lysates (Fig. 9b, lanes 5–8). This result is in line with the finding that Pol was not detectable in any of the particles containing myr-Gag (Fig. 9b, left hand panel), suggesting a failure to incorporate Pol into capsids. While reduced budding of the myr-Gag mutants (Src-mLQQLY, Src-mHGDII, Src-mPGDRW) may interfere with Pol detection in particles, Pol was also not detected in Src-Gag particles with only modestly reduced budding. Analysis of cell lysates showed that Pol was expressed at comparable amounts by all constructs (Fig. 9b, right panel).

TEM studies of selected samples showed that in cells producing fully infectious wt FFV particles and cells expressing wt-Gag proteins, cytosolic capsids are abundant (Fig. 9c, left and middle panels labelled FFV and Gag SVP) while myr-Gag assemblies were highly associated with intracellular membranes or present in small membranous vesicles (Fig. 9c, right panels labelled Myr-Gag-SVP). These particles were often irregular in shape and clearly distinct from Gag-only cytosolic capsids (Fig. 9c, middle panel labelled Gag SVP) or released wt particles. In addition to intracellular budding structures, myr-Gag accumulated at the plasma membrane, leading to the formation of budding structures and Gag SVPs. As expected, none of the wt or mutant myr-Gag genomes were infectious (data not shown).

**Fig. 10:**
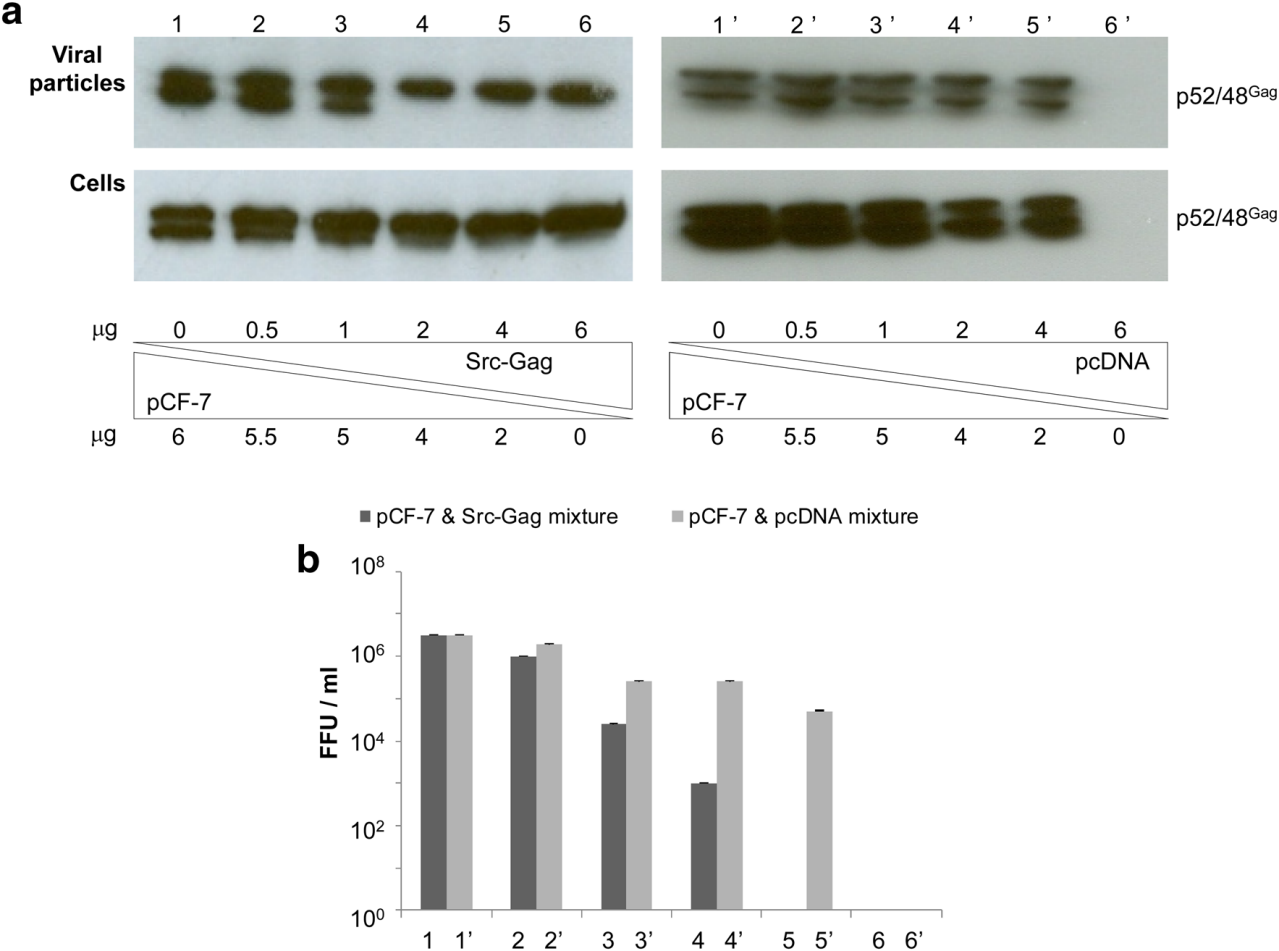
Myristoylated Gag interferes with Gag processing and viral titer in a trans-dominant negative fashion. **a** 293T cells were co-transfected with different ratios of the FFV wt genome pCF-7 and the proviral Src Gag clone (*lanes 1–6*) or the empty vector pcDNA (*lanes 1′–6′*). Cell lysates and VLPs in supernatants were analyzed by SDS-PAGE. FFV Gag p48 and p52 are marked. **b** Two days p.t., infectivity was determined by titration of culture supernatants on FeFab cells. Mean titers of three independent experiments are given. *Error bars* represent the standard deviation

To determine whether co-expression of myr-Gag interferes with wt FFV replication or, alternatively, even increases infectious viral particle release, 293T cells were co-transfected at different ratios (6:0, 5.5:0.5, 5:1, 4:2, 2:4, 0:6) of wt to myr-Gag-expressing proviral plasmids pCF-7 and Src-Gag. As a background control, Src-Gag was replaced by pcDNA. Co-transfection of pCF-7 and pcDNA showed only a modest, dose-dependent decline in viral budding and infectivity (Fig. 10a, lanes 1′–6′). In contrast, beginning at a 5:1 ratio of wt FFV to Src-Gag genomes, Gag processing clearly decreased in cell lysates and released particles (Fig. 10a, lanes 1–6). Concomitantly, titers drastically declined with a decreasing wt to Src-Gag ratio confirming the trans-dominant negative effect of Src-Gag variants (Fig. 10b). Infectivity and Gag processing were completely abolished when Src-Gag-encoding proviruses exceeded one-third of the total input.

### Comparison to simian foamy virus structure

**Fig. 11:**
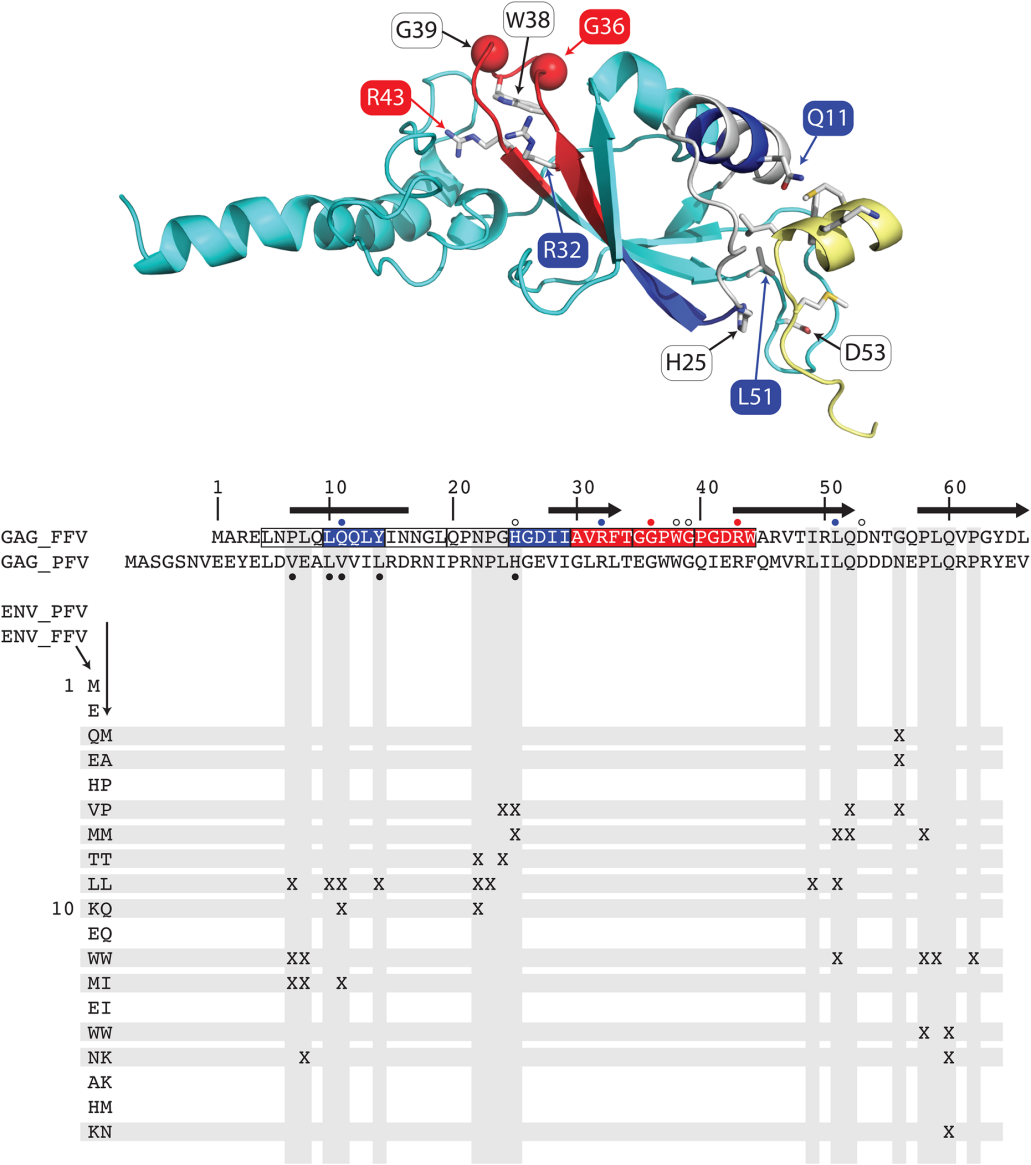
Mutations at the Gag–Elp interface or central beta-sheet affect budding and Gag folding. Modelled structure of the FFV Gag–Elp interaction indicates that mutations affecting only budding tend to localize at the Gag–Elp interface. Mutations which also affect Gag processing and capsid assembly localize to the central beta-sheet, therefore likely affecting the overall folding of Gag. The *top panel* shows a structure of the interaction of FFV Gag (*cyan ribbon*) and Env (*yellow ribbon*) modelled using the PFV structure [38] as a template (only one dimer of the dimer–dimer is shown). The eight 5-mers mutated to alanine are *colored* by phenotype (*white*, wild-type; *blue*, no budding; *red*, no budding, Gag processing or capsid assembly). Side chains of single point mutations in Gag are labelled and shown as *sticks colored* by atom type (except for glycine C-alpha atoms, shown as spheres). Labels are *colored* by phenotype. Env side chains interacting with Gag are shown as *sticks colored* by atom type. Image was created with PyMOL [http://sourceforge.net/projects/pymol/]. The *bottom panel* shows protein sequence alignments of FFV and PFV Gag and a matrix of. Inter-molecular side-chain interactions indicated with ‘X’. Helical regions identified from the human Gag structure are shown as a *black bar*; beta strands are shown as a *black arrow*. Sequence numbers refer to the FFV sequence. The 5-mer mutants and the single point mutants are colored by phenotype as above (GAG_FFV *boxes* and *dots*, respectively). Gag residues playing a role in Gag-Env affinity or particle egress [38, 57] are indicated with *black dots*

The structure of the FFV Gag-Env interaction (Fig. 11, modelled on the PFV structure [38]) indicates that mutations that affect only budding tend to be located at the Gag-Env interface (Gag Q11 and L51). Conversely, mutations which also affect Gag processing and capsid assembly (G36 and R43) localize to the central beta-sheet and are therefore likely to affect the folding of the Gag structure (Fig. 11 and Additional file 3: Table S1). Gag R32 also resides in the central beta-sheet but only affects budding, indicating that the structural rearrangement here is likely to be more subtle. Gag H25A and D53A, which have an attenuated or wt phenotype, respectively, also occur in or near the Gag-Env interface but on Gag loops interacting with Env loops, both of which may be flexible (Fig. 11 and Additional file 3: Table S1).

## Discussion

Here, we present data on the functional importance of the N-terminus of FFV Gag for capsid formation, Gag processing, particle budding and infectivity. The data confirm and expand the current literature on FV morphology and assembly, based mainly on studies in PFV [24, 39]. However, our results also highlight significant differences between PFV and FFV. Comparison of PFV and non-primate FVs reveals that the N-terminus of PFV Gag has seven extra, mostly non-essential residues (Fig. 4a). Furthermore, PFV Gag also contains a predicted coiled-coil region of up to 130 additional amino acids, a feature visible by comparative cryo-electron microscopy of FFV and PFV particles [14, 28, 30].

Alanine scanning mutagenesis of FFV Gag residues 5–9 (LNPLQ) slightly impaired particle release but led to a 1000-fold decrease in titer, strongly indicating that this region is essential for infection of new host cells but not assembly, processing or release. Meanwhile, residues 10–14 are involved in Gag protein processing, particle release and infectivity. Specifically, though the FFV Gag L10A mutant was still capable of proper capsid formation and infectious particle release, the PFV Gag L17A mutant had dramatically reduced viral titers and PFV Gag L17S was even unable to release particles [24]. In contrast, a mutation of the neighboring residue, FFV Gag Q11A, allowed normal Gag processing and capsid assembly, but abolished Elp interaction, impacting particle release and infectivity, pointing to Q11’s essential function in budding.

Alanine mutations of an extended, modestly conserved N-terminal region of FFV Gag (residues 15–24, INNGL and QPNPG) led to wt phenotypes, despite a substantial increase in hydrophobicity. In PFV Gag, this region forms an exposed loop (PFV residues 22–31; Fig. 11 [38]), and our data suggest that the precise conformation is not important for function. However, substitution of the same 10-mer sequence (INNGLQPNPG) by five, eight or ten alanines showed that the latter two substitutions yielded a wt phenotype with a slight reduction in titer whereas replacement of the 10-mer sequence by only five alanines led to wt levels of particle budding and Gag processing but a 100-fold decrease in titer (data not shown). This indicates that the spacing of critical elements is essential for proper conformation and function of the N-terminus of Gag, at least during target cell infection.

Alanine replacements of FFV Gag residues 25–29 (HGDII) completely blocked budding but did not affect capsid formation and Gag processing, suggesting a defect in Elp interaction or capsid transfer to the site of budding. Of the five exchanges, only H25A impaired but not complete blocked budding and infectivity, similar to the PFV Gag H32 mutant [24]. In contrast, alanine replacement of each of the subsequent 5-mer blocks (AVRFT, GGPWG and PGDRW) fully abrogated capsid assembly, Gag processing, particle release and infectivity. In PFV Gag, this region forms part of a beta-sheet and mutations likely lead to aberrant protein folding [24, 32].

Mutation of the highly conserved FFV Gag CTRS residue R43 cripples capsid formation, Gag processing and particle release, similar to mutation of PFV Gag residue R50 [11, 39]. In contrast, FFV Gag mutant W38A has a wt phenotype, while PFV Gag W45A is defective in particle release and replication.

Particle budding, Elp interaction and infectivity are abrogated by FFV Gag mutations Q11A, and L51A (Figs. 4, 7) while capsid formation is not affected. The structure of the FFV Gag–Elp interaction (Fig. 11, modelled on the PFV structure [38]) indicates that mutations affecting only budding (Q11A, H25A, and L51A, Figs. 4 and 7) tend to localize to the Gag–Elp interface, implying that accessibility of these residues is required for Gag–Elp interaction. In contrast, mutations also affecting capsid assembly and Gag processing (G36 and R43) may hinder folding of the central beta-sheet and distort the overall structure. Gag assembly therefore mainly requires a defined structure and proper folding of the Gag N-terminus.

The current study also explores the sub-cellular site of capsid assembly and RNA and Pol packaging. Three important features were observed when targeting FFV capsid assembly to intracellular membranes and the plasma membrane using myr signals. Firstly, low-level budding of unprocessed Gag was observed, even by the assembly-deficient Gag CTRS mutant mPGDRW, similar to PFV [11]. This indicates that membrane targeting can at least partially overcome a budding defect or that Gag contains additional domains important for assembly. Secondly, retargeted FFV Gag exhibited a nearly complete absence of cell-associated processing, even in the presence of the wt virus. Under similar conditions, PFV was still capable of reduced levels of Gag processing [11]. Thirdly, co-transfection of wt and myr-Gag proviruses showed that the myr signal was clearly *trans*-dominant over the N-terminal MTOC-targeting region in Gag (Fig. 10).

RNA packaging and concomitant Pol incorporation likely occur in the cytoplasm around the MTOC [40]. Thus, assembly site retargeting to membranes via a myr signal may yield empty viral cores lacking genomes or Pol since capsids do no longer co-localize with the viral genome and Pol detectable by the inability of these mutants to process Gag. C-terminal cleavage of wt FFV Gag was dramatically suppressed by myr-Gag, even at a wt:myr-Gag ratio of 5:1. Alternatively, the aberrant capsid structures seen in TEM images (Fig. 9c) may not allow genome and Pol packaging, again leading to a defect in Gag processing.

Importantly, our data show a clear correlation between FV capsid assembly and Gag processing (see Additional file 3: Table S1), potentially homologous to budding-induced Gag and Gag-Pol processing of orthoretroviruses [1]. The data for FFV provided here show a clear correlation between defects in cytosolic capsid assembly and the absence of terminal Gag processing in mutants mAVRFT, mGGPWG, mPGDRWT, G36A and R43A. Cartellieri et al. [39] detected wt intracellular Gag processing by the assembly-deficient PFV R50A mutant. In contrast, Eastman and Linial [11] reported reduced Gag processing by PFV R50A and R50 W mutants, similar to our results with the corresponding FFV Gag mutant.

In addition to assembly-competent Gag and the full-length FFV Pol precursor, Gag processing also requires FFV genome packaging into virus particles (Fig. 8). While a packaging-competent proviral genome is absolutely required, the Env-encoded particle budding machinery is fully dispensable. This finding fits well into the current model of FV Pol incorporation, where viral RNA genome encapsidation during or after cytosolic capsid assembly also mediates Pol packaging via binding to the Pol encapsidation signal on the genome [10, 21]. Reminiscent to MTOC targeting of Gag for capsid assembly in FVs and B/D type retroviruses, genomic RNA of Mason-Pfizer monkey virus, which also assembles at the MTOC, is directed to the site of assembly by microtubule binding [41]. Conversely, HIV genomes are evenly distributed throughout the cell, allowing genome packaging at the plasma membrane, the site of assembly [41]. It is currently unknown but likely that FV genomes are also targeted to the MTOC for efficient genome encapsidation.

While FV Gag processing depends on capsid formation but is clearly budding-independent as shown in Fig. 8, this is not the case for FV Pol auto-processing. FV Pol is expressed independently of Gag via a spliced transcript and undergoes fundamentally different activation mechanisms compared to orthoretroviruses [18, 42, 43]. As shown here for FFV and previously for PFV, Pol undergoes auto-processing independent of co-expressed Gag, though part of the *pol* transcript may be essential for PR dimerization and processing [19, 43].

Using Pol packaging and terminal Gag cleavage as surrogate markers for genomic RNA incorporation, the mutations introduced at the 5′-end of the *gag* gene did not affect the 5′ genomic RNA encapsidation signal, as released particles contained processed Pol and cleaved Gag (Figs. 2b, 4b, 8). Though PFV Pol is freely released from cells [44], our data show that FFV Pol is released only in budded particles (Fig. 8). Previous studies suggest that PFV Gag GR box 1 mutants, which package genomes into particles, do not encapsidate Pol, likely due to abrogation of essential Gag-Pol interactions, indicating that genome binding to Pol is insufficient [45].

In summary, our study identifies specific residues in FFV Gag essential for capsid assembly and Elp interaction. We found a clear correlation between FV capsid assembly and Gag processing, homologous to budding-induced Gag and Gag-Pol processing observed in orthoretroviruses. Finally, genome-dependent incorporation of Pol into capsids appears to be dependent on the site of assembly and is a prerequisite for Gag processing. Our data indicate a central role of the N-terminus of Gag in at least three completely different functions: FV assembly, maturation and budding. The data show that FV Gag processing occurs independent of particle budding, a mechanism distinct from that used by most other retroviruses.

## Conclusions

Foamy viruses have developed a unique replication strategy combining the classical type B/D retroviral capsid assembly pathway and an FV-specific Env-dependent particle budding process. The determinants of foamy virus (FV) Gag essential for interaction with the Env leader protein Elp, a stable and particle-associated N-terminal Env-derived protein, and the multiple functions of the N-terminal region of prototype/primate FV (PFV) Gag during viral assembly and budding still remain poorly understood. Here, we show that residues essential for interaction with Elp, particle budding, and release are located in the N-terminus of Gag. Distinct, more centrally positioned residues within the cytoplasmic targeting-retention signal are essential for capsid formation, as determined by sedimentation assays. Furthermore, we found that genomic FFV RNA packaging is essential for Gag processing by the Pol-encoded protease PR. These findings provide new insights into the functional interaction and coordination of different FV structural components during assembly in the FV life cycle.

## Methods

### Cell culture, DNA transfection and indirect immunofluorescence

FeFab (created in our lab using Crandell feline kidney cells, CrFK, ATCC, Manassa, USA; see [46]), Hela and 293T (both from ATCC) cells were grown in Dulbecco’s modified Eagle’s medium (Sigma-Aldrich) supplemented with 10 % fetal calf serum (PAN Biotech) and 1 % penicillin–streptomycin (Sigma-Aldrich), respectively [32, 46, 47]. Cell identity and absence of contaminants was confirmed by multiplex PCR-based cell typing and pathogen detection performed by Multiplexion, Heidelberg. Transfection of sub-confluent 293T cells with plasmid DNA was performed by calcium co–precipitation on in 6 or 10 cm dishes as described previously [48]. Cells and cell culture supernatants were harvested two d p.t. Indirect immunofluorescence of paraformaldehyde-fixed transfected HeLa cells allowing a much better analysis of subcellular localization than the 293T cells using the FFV Gag MA antiserum was done as previously described [32].

### Titration of FFV and FFV-related viral vectors using FeFab cells

FeFab cells [46] were seeded in 96-well plates (3 × 10^4^ cells in 100 μl culture medium per well). Four hours (h) post-seeding, supernatants containing wild-type (wt) FFV or FFV variants were cleared by centrifugation at 405×*g* for 5 min at room temperature. Supernatants were serially diluted five-fold by mixing 25 μl viral supernatant into the first well then transferring 25 μl diluted supernatant into subsequent wells. Each sample was titrated in duplicate. At 48 h post-infection (p.i.), cells were fixed and stained as described previously [46]. Titers were calculated by multiplying the number of blue-stained nuclei multiplied by the highest dilution factor.

### Purification of FFV particles and virus-like particles (VLPs)

Cell culture supernatants (5 ml) of transfected 293T cells were cleared of cellular debris by centrifugation (10 min, 405×*g*). FFV particles or VLPs were pelleted through a 2 ml cushion of 20 % sucrose in PBS (w/v) by ultracentrifugation in a SW41Ti rotor (Beckman Coulter, Krefeld, Germany) (2 h, 100,000×*g*, 4 °C). The invisible pellet, devoid of medium and sucrose, was re-suspended in 50 μl of 1 % SDS in PBS containing complete protease inhibitor cocktail (Roche) and stored at −20 °C before immunoblotting.

### Sucrose gradient sedimentation analysis of intracellular capsids

Sucrose gradient centrifugation of intracellular capsids was performed with modifications to a method described previously [11]. Transfection of 293T cells was performed in 10 cm dishes. At 36 h p.t., cells were washed three times with ice-cold PBS and scraped using a rubber policeman. Cells detached as thin white sheets were transferred into 15 ml conical tubes. Cells were pelleted by centrifugation (5 min, 405×*g*). Pellets were resuspended in 0.5 ml lysis buffer (10 mM Tris–HCl, pH 7.4, 5 mM MgCl_2_, 100 mM KCl, 2 mM dithiothreitol, EDTA-free complete protease inhibitor cocktail) without Triton X-100 and transferred into 1.5 ml Eppendorf tubes. An equal volume of lysis buffer with 0.2 % Triton X-100 was added to each tube and mixed gently. Pellets were homogenized by shearing five times with an ice-cold 26-gauge syringe cleared of air bubbles. After shearing, the cell pellet was left on ice for 10 min, then sedimented for 10 min at 4 °C at 1300×*g* in a pre-cooled Eppendorf centrifuge. Supernatants of cytoplasmic extracts containing the particles were carefully loaded onto a preformed and stepwise sucrose gradient consisting of 600 μl of 10, 20, 30, 40, 50, and 60 % sucrose in 1× PBS equilibrated overnight. Gradients were centrifuged in a SW60Ti rotor (Beckman Coulter, 1 h, 35,000 rpm, 4 °C). Particles migrate through the gradient corresponding to their sedimentation behavior. A total of six fractions (700 μl each) were collected from the top of the gradient using a 1 ml micropipette and stored at −70 °C before analysis by immunoblotting.

### Molecular cloning of FFV constructs

FFV vector pCF-7-ubi-lacZ-mATG (mATG), containing an inactivated *gag* ATG, and the RNA-packaging deficient vectors pCF-7-ubi-lacZ-mATG-GC (mATG-GC), pCF-7-ubi-lacZ-mΔBBBB-GC (mΔBBBB-GC) and pCF-EO-GC (EO-GC) were recently described [35]. pMP-Pol-oPRE was used for FFV Pol expression. The cytomegalovirus immediate-early promoter-based Gag expression clone pBC-Gag-oPRE and the infectious FFV molecular clone pCF-7 were used as cloning backbones [35, 49].

PCR-mediated mutagenesis was used to introduce different small N-terminal Gag deletions into pCF-7. PCR sense primers E4AΔ5, E4AΔ7, E4AΔ9, E4AΔ11 and antisense primer AS (Additional file 4: Table S2) were used. The amplicons were digested with XhoI and BlpI and inserted into the correspondingly digested pCF-7. The new constructs are designated E4AΔ5, E4AΔ5-7, E4AΔ5-9 and E4AΔ5-11, respectively.

Alanine scanning mutagenesis was performed via fusion PCR [50] to systematically replace blocks or defined N-terminal residues by alanines (A). The Gag constructs and corresponding primers are designated with the residue(s) substituted by alanine(s), For example, mLNPLQ indicates a replacement of the LNPLQ motif by 5 alanines. Likewise, R43A indicates a replacement of FFV Gag arginine-43 by alanine (Additional file 4: Table S2). Corresponding regions of each gene were amplified by PCR. For example, to clone mutant Gag mLNPLQ, the upstream fragment was amplified using primers dLNPLQ-1s and dLNPLQ-1as; the downstream fragment was amplified using primers dLNPLQ-2s and dLNPLQ-2as (Additional file 4: Table S2). Both PCR products were fused in a third PCR using primers Gag mut-1s and Gag mut-2as. The resulting amplicons were cloned into pCF-7 or pBC-Gag-oPRE using XhoI and SmaI.

To create proviral myr-Gag mutants, the nine-amino acid src signal sequence was engineered onto the N-terminus of wt or mutant Gag (mLQQLY, mHGDII, mPGDRW) in pCF-7. The complementary oligonucleotides Src(+) and Src(−), containing the src signal (Additional file 4: Table S2), were annealed, generating SwaI and XhoI overhangs. The annealed oligonucleotides were cloned into pCF-7 using SwaI and XhoI, generating the corresponding mutants Src-Gag, Src-mLQQLY, Src-mHGDII and Src-mPGDRW, respectively.

### Expression of his-tagged FFV Elp in *Escherichia coli* BL21

The FFV N-terminal leader protein Elp was recombinantly produced in *E. coli* BL21 as an N-terminal His-tag fusion protein [14]. Pre-cultures of 200 ml LB medium containing 50 µg/ml ampicillin were inoculated from glycerol stocks of *E. coli* BL21 transformed with the pET32a(+)-His-Elp expression plasmid and grown at 37 °C and 200 rpm overnight. Overnight cultures were used to inoculate 1 l of LB medium containing 50 µg/ml ampicillin in a 5 l flask. Cultures were grown at room temperature under shaking conditions till an OD_600_ of 0.6. Expression of His-tagged fusion proteins was induced by the addition of IPTG at a final concentration of 250 μM. Protein expression cultures were grown for a further 6 h under the same conditions before harvesting at 8000 rpm and 4 °C for 10 min in a Beckmann Sorvall centrifuge using the SLA300 rotor. Pellets were resuspended in 5 ml 1× PBS supplemented with 1× protease inhibitor and 2 mM DTT and stored at −20 °C. To prepare cleared His-Elp lysates under native conditions, *E. coli* pellets were thawed on ice for 15 min and disrupted by passing the cell suspension three times through an Emulsiflex C5 French press (AVESTIN) at 1000–1500 bars. Cell lysates were cleared at 10,000×*g* for 30 min at 4 °C. Cleared supernatants containing the soluble proteins were stored on ice for pull-down assays.

### Immunoblot analyses

Transfected 293T cells in 10 cm dishes were harvested 2 days p.t. and lysed in 1 % SDS. Immunoblotting was performed as previously described [30]. Expression of Gag, Env and Pol in cell lysates and released particles were visualized using guinea pig polyclonal serum against the capsid CA domain of FFV Gag (α-FFV CA, 1:2000) [51], goat polyclonal serum against the TM domain of FFV Env (α-FFV TM, 1:1000 dilution) and rabbit polyclonal serum against the protease domain of FFV Pol (α-FFV PR’ 1:3000) in 5 % milk powder in PBS containing 0.1 % Tween-20. After incubation with secondary antibody conjugated with horse radish peroxidase (HRP, 1:5000), the membranes were developed using ECL™ western blotting detection reagents (GE Healthcare). Amersham Hyperfilm™ ECL films (GE Healthcare) were exposed to the resulting chemiluminescence signals for 5 s to 3 min before development in an AGFA film processor.

### Pull-down assay

Transfected 293T cells in 10 cm dishes were harvested 2 days p.t. Cells were washed once with 1× PBS and lysed in cold 600 μl Triton lysis buffer (TLB, 20 mM Tris, 137 mM NaCl, 10 % glycerol, 100 mM KCl, 1 % Triton X-100, pH 7.4) supplemented with 1× EDTA-free protease inhibitor and transferred to a sterile 1.5 ml Eppendorf tube as described previously [52]. Cells were lysed at 4 °C for 1 h in an overhead shaker. Meanwhile, Ni–NTA agarose (Qiagen) was prepared by transferring 100 μl of Ni–NTA slurry (50 μl bed volume) to a 1.5 ml Eppendorf tube and washing three times with 1 ml cold PBS and twice with 1 ml TLB.

Equilibrated Ni–NTA agarose were carefully sedimented after centrifugation for 10 min at 500×*g* and mixed with 1 ml cleared *E. coli* lysates containing His-tagged recombinant proteins on a rotary shaker (200 rpm, 4 °C, 60 min). *Escherichia coli* lysate-Ni–NTA mixtures were sedimented to remove the supernatant and washed three times in cold PBS for 10 min at 400×*g*. Cell lysates (500 μl) were added to the mixture and shaken on a rotary shaker (200 rpm, 4 °C, 60 min). After washing three times in cold TLB for 10 min at 400×*g*, His-tagged Elp was eluted from the Ni–NTA agarose by adding 60 μl 1 % SDS and boiling the samples at 95 °C for 5 min. Ni–NTA agarose was sedimented by centrifugation for 10 min at 15,000 rpm. Supernatants were transferred to a new 1.5 ml Eppendorf tube. Aliquots of cell lysates and eluates were analyzed by SDS-PAGE and immunoblotting. Elp-His was detected with a polyclonal antibody against the His-tag (Abcam). FFV Gag was detected with an α-FFV CA polyclonal serum (described above).

### Transmission electron microscopy analysis

For transmission electron microscopy (TEM) 293T cells were transfected in 6 cm dishes with 6 μg of plasmid DNA. At 2 days p.t., cells with a transfection efficiency greater than 60 % were washed three times and fixed in situ with 2.5 % glutaraldehyde in 50 mM sodium cacodylate (pH 7.6) for 30 min at 4 °C before processing as described previously [53].

### Genomic RNA extraction from viral particles and qRT-PCR

Genomic RNA in viral particles was extracted using Trizol^®^ (Invitrogen) according to the manufacturer’s instructions. RNA was incubated with 2 U of RNase-free DNase I (New England Biolabs). RNA was converted to cDNA using the QuantiTect Reverse Transcription Kit (Qiagen) using the cDNA synthesis primer 5′-GTAGGTGTGCGGTAGGCTTT-3′. qPCR of a ubiquitinC promoter fragment encoded by the vector genomes was performed using SYBR Green and the cDNA synthesis primer and sense-primer 5′-CTGACGCCTCACTTATCCCT-3′ as described previously [35]. cDNA was used for quantitative RT-PCR analysis.

### Modelling of Gag-Env interaction structure

The structure of the interaction between foamy virus GAG and ENV was modelled using Modeller [54] with the human structure [PDB 4jmr, 38] as a template. Contacts between side-chains were identified using RasMol [55].

## Additional files

10.1186/s12977-016-0291-8 Defined alanine substitution mutations of N-terminal Gag sequence inhibit syncytia formation in transfected 293T cells. 293T cells were transfected in 6 cm dishes with 6 μg of plasmids expressing proviral Gag mutants (mLNPLQ, mLQQLY, mINNGL, mQPNPG, mHGDII, mAVRFT, mGGPWG and mPGDRW, panel **A**) or (Q11A, H25A, R32A, G36A, W38A, G39A, R43A, L51A, D53A, panel **B**), pCF-7, or pCDNA using calcium phosphate. Transfection efficiency was monitored by co-transfection of 1 μg pGfp. At 12 hrs p.t., cells were visualized by Gfp auto-fluorescence. Bright GFP-positive individual cells as well as large areas representing fused neighboring cells transfected by fusion-competent mutants are detectable.
10.1186/s12977-016-0291-8 Subcellular localization of five alanine scanning Gag mutants. Hela cells were transfected with proviral pCF-7-based Gag mutants (mLNPLQ, mLQQLY, mINNGL, mQPNPG, mHGDII, mAVRFT, mGGPWG, or mPGDRW) or the parental provirus pCF-7. pcDNA plasmid was used for transfection as negative control. Thirty-six hours post-transfection, cells were fixed and stained with a rabbit polyclonal antiserum generated against FFV Gag and Alexa-488-conjugated secondary antibody. Nuclei were stained with DAPI. Scale bars are 50 µm in length.
10.1186/s12977-016-0291-8 Summary of single amino acid mutagenesis of defined N-terminal FFV Gag residues. Comparative characterization of wt and Gag mutant FFV proviruses with respect to FFV infectivity, particle formation and budding, Elp interaction, Gag processing and syncytia formation. Phenotypes are characterized as +++: like wt; +: strongly reduced; - negative/absent.
10.1186/s12977-016-0291-8 Primers used for cloning and site-directed mutagenesis. Underlined characters indicate a SwaI single-stranded overhang. Bold text indicates an XhoI restriction site.

## Abbreviations

BFV: bovine foamy virus
CC: coiled-coil
CTRS: cytoplasmic targeting and retention signal
EFV: equine foamy virus
Elp: Env leader protein
FFV: feline foamy virus
FV: foamy virus
HIV: human immunodeficiency virus
IN: integrase
MTOC: microtubule organizing center
PFV: prototype/primate foamy virus
p.t.: post-transfection
wt: wild-type
