# Supplementary figures and images for: Mutagenesis of N-terminal residues of feline foamy virus Gag reveals entirely distinct functions during capsid formation, particle assembly, Gag processing and budding
Source: Retrovirology. 2016 Aug 22;13(1):57. doi: 10.1186/s12977-016-0291-8 (PMC4994201; doi:10.1186/s12977-016-0291-8)

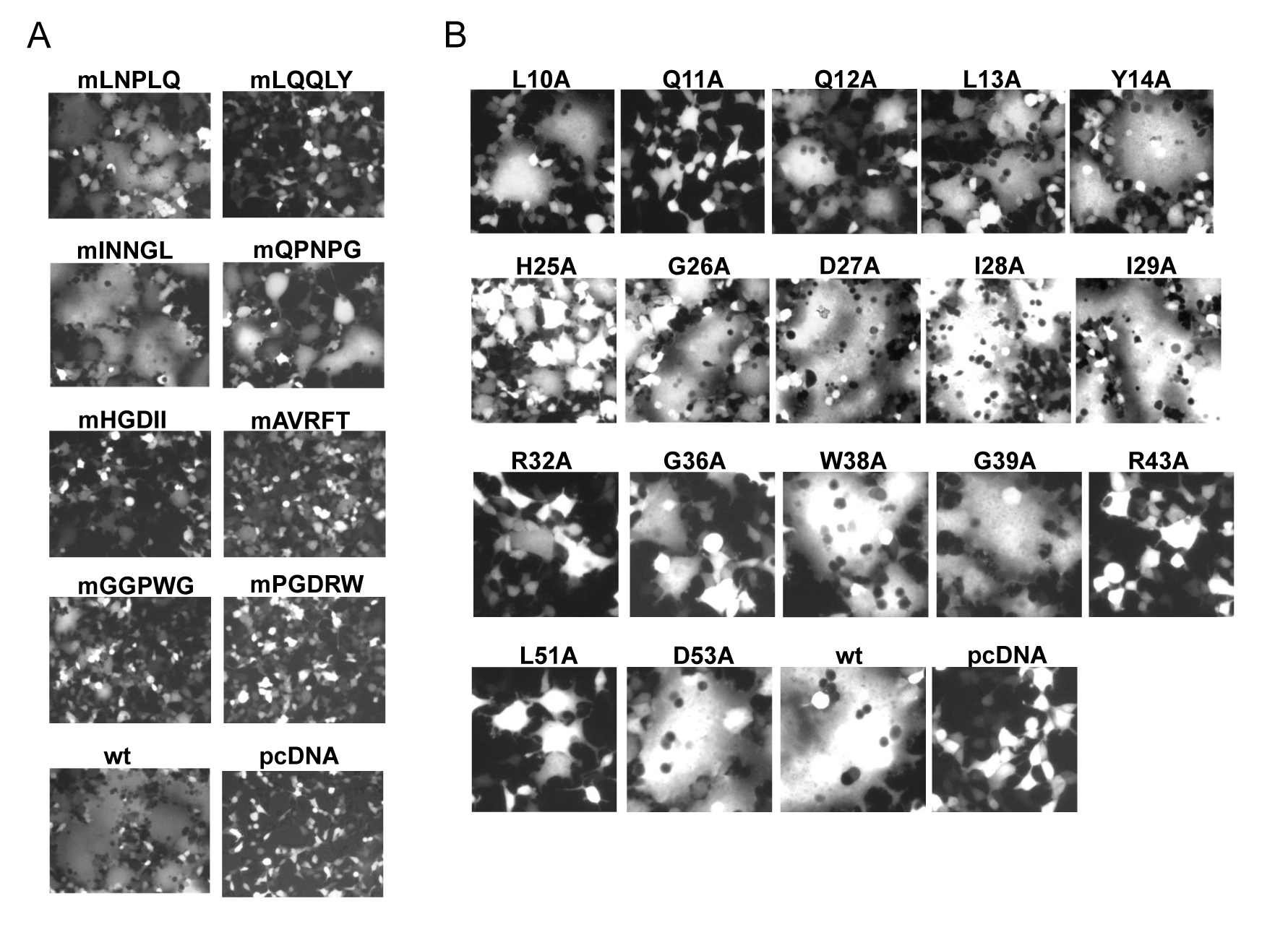

Supplement: Supplementary file 1 — 10.1186/s12977-016-0291-8 Defined alanine substitution mutations of N-terminal Gag sequence inhibit syncytia formation in transfected 293T cells. 293T cells were transfected in 6 cm dishes with 6 μg of plasmids expressing proviral Gag mutants (mLNPLQ, mLQQLY, mINNGL, mQPNPG, mHGDII, mAVRFT, mGGPWG and mPGDRW, panel A) or (Q11A, H25A, R32A, G36A, W38A, G39A, R43A, L51A, D53A, panel B), pCF-7, or pCDNA using calcium phosphate. Transfection efficiency was monitored by co-transfection of 1 μg pGfp. At 12 hrs p.t., cells were visualized by Gfp auto-fluorescence. Bright GFP-positive individual cells as well as large areas representing fused neighboring cells transfected by fusion-competent mutants are detectable. [file 12977_2016_291_MOESM1_ESM.tif]

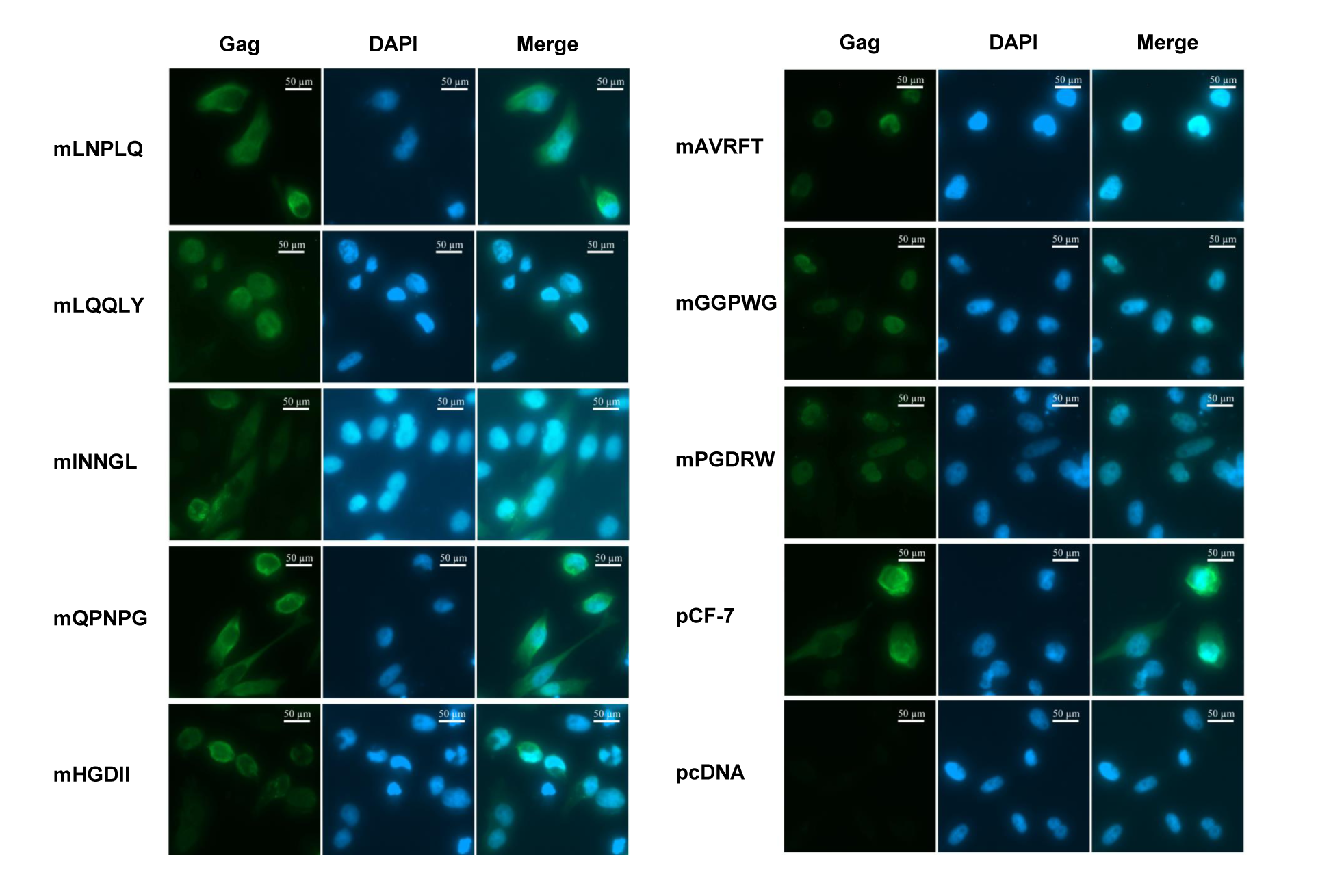

Supplement: Supplementary file 2 — 10.1186/s12977-016-0291-8 Subcellular localization of five alanine scanning Gag mutants. Hela cells were transfected with proviral pCF-7-based Gag mutants (mLNPLQ, mLQQLY, mINNGL, mQPNPG, mHGDII, mAVRFT, mGGPWG, or mPGDRW) or the parental provirus pCF-7. pcDNA plasmid was used for transfection as negative control. Thirty-six hours post-transfection, cells were fixed and stained with a rabbit polyclonal antiserum generated against FFV Gag and Alexa-488-conjugated secondary antibody. Nuclei were stained with DAPI. Scale bars are 50 µm in length. [file 12977_2016_291_MOESM2_ESM.tif]
