# Supplementary material for: Mutagenesis of N-terminal residues of feline foamy virus Gag reveals entirely distinct functions during capsid formation, particle assembly, Gag processing and budding
Source: Retrovirology. 2016 Aug 22;13(1):57. doi: 10.1186/s12977-016-0291-8 (PMC4994201; doi:10.1186/s12977-016-0291-8)
Supplement: Supplementary file 3 — 10.1186/s12977-016-0291-8 Summary of single amino acid mutagenesis of defined N-terminal FFV Gag residues. Comparative characterization of wt and Gag mutant FFV proviruses with respect to FFV infectivity, particle formation and budding, Elp interaction, Gag processing and syncytia formation. Phenotypes are characterized as +++: like wt; +: strongly reduced; - negative/absent. [file 12977_2016_291_MOESM3_ESM.docx]

Table S1. Summary of single amino acid mutagenesis of defined N-terminal FFV Gag residues^a^

|  | **Relative Infectivity** | **Capsid Assembly** | **Gag Processing** | **Particle Budding** | **Elp Interaction** | **Syncytia Formation** |
| --- | --- | --- | --- | --- | --- | --- |
| **wt** | **+++** | **+++** | **+++** | **+++** | **+++** | **+++** |
| **Q11A** | **-** | **+++** | **+++** | **-** | **-** | **-** |
| **R32A** | **-** | **+++** | **+++** | **-** | **-** | **+** |
| **G36A** | **-** | **-** | **-** | **-** | **-** | **+** |
| **R43A** | **-** | **-** | **-** | **-** | **-** | **-** |
| **L51A** | **-** | **+++** | **+++** | **-** | **-** | **+** |

^a^ Comparative characterization of wt and Gag mutant FFV proviruses with respect to FFV infectivity, particle formation

and budding, Elp interaction, Gag processing and syncytia formation. Phenotypes are characterized as +++: like wt;

+: strongly reduced; - negative/absent.
