# Supplementary material for: Mutagenesis of N-terminal residues of feline foamy virus Gag reveals entirely distinct functions during capsid formation, particle assembly, Gag processing and budding
Source: Retrovirology. 2016 Aug 22;13(1):57. doi: 10.1186/s12977-016-0291-8 (PMC4994201; doi:10.1186/s12977-016-0291-8)
Supplement: Supplementary file 4 — 10.1186/s12977-016-0291-8 Primers used for cloning and site-directed mutagenesis. Underlined characters indicate a SwaI single-stranded overhang. Bold text indicates an XhoI restriction site. [file 12977_2016_291_MOESM4_ESM.docx]

**Table S2. Primers used for cloning and site-directed mutagenesis.**

| **Name** | **Sequence (5’-3’)** |
| --- | --- |
| **Alanine mutagenesis of the N-terminus of FFV Gag** | |
| dLQQLY-1as | GGCAGCGGCAGCGGCTTGGAGAGGATTTAATTCTCG |
| dLQQLY-2s | GCCGCTGCCGCTGCCATAAATAATGGCTTACAACC |
| dINNGL-1as | GGCAGCGGCAGCGGCATACAGTTGCTGTAATTGGAG |
| dINNGL-2s | GCCGCTGCCGCTGCCCAACCTAATCCAGGACATGGAG |
| dQPNPG-1as | GGCAGCGGCAGCGGCTAAGCCATTATTTATATACAGTTG |
| dQPNPG-2s | GCCGCTGCCGCTGCCCATGGAGATATTATTGCTGTC |
| ddHGDII-1as | GGCAGCGGCAGCGGCTCCTGGATTAGGTTGTAAGCC |
| dHGDII-2s | GCCGCTGCCGCTGCCGCTGTCAGATTTACAGGAGGAC |
| dAVRFT-1as | GGCAGCGGCAGCGGCAATAATATCTCCATGTCCTGG |
| dAVRFT-2s | GCCGCTGCCGCTGCCGGAGGACCTTGGGGTCCAGGTG |
| dGGPWG-1as | GGCAGCGGCAGCGGCTGTAAATCTGACAGCAATAATATC |
| dGGPWG-2s | GCCGCTGCCGCTGCCCCAGGTGATAGATGGGCTAGAGTG |
| dPGDRW-1as | GGCAGCGGCAGCGGCACCCCAAGGTCCTCCTGTAAATCTG |
| dPGDRW-2s | GCCGCTGCCGCTGCCGCTAGAGTGACAATACGATTACAAG |
| R32A-2s | ATTGCTGTCGCCTTTACAGGAGGACCTTGG |
| R32A-1as | CCTGTAAAGGCGACAGCAATAATATCTCCATGTC |
| G36A-2s | TTACAGGAGCCCCTTGGGGTCCAGGTGATAG |
| G36A-1as | GACCCCAAGGGGCTCCTGTAAATCTGACAGC |
| W38A-2s | GAGGACCTGCCGGTCCAGGTGATAGATGG |
| W38A-1as | CACCTGGACCGGCAGGTCCTCCTGTAAATCTGACAG |
| G39A-2s | GAGGACCTTGGGCCCCAGGTGATAGATGGGCT |
| G39A-1as | CACCTGGGGCCCAAGGTCCTCCTGTAAATCTG |
| R43A-2s | CCAGGTGATGCCTGGGCTAGAGTGACAATACG |
| R43A-1as | TCTAGCCCAGGCATCACCTGGACCCCAAGG |
| L51A-2s | CAATACGAGCCCAAGATAACACAGGACAACC |
| L51A-1as | GTGTTATCTTGGGCTCGTATTGTCACTCTAGCC |
| D53A-2s | CGATTACAAGCCAACACAGGACAACCTTTAC |
| D53A-1as | TCCTGTGTTGGCTTGTAATCGTATTGTCACTCTAGC |
| L10A-2s | CCTCTCCAAGCCCAGCAACTGTATATAAATAATG |
| L10A-1as | CATTATTTATATACAGTTGCTGGGCTTGGAGAGG |
| Q11A-2s | CCTCTCCAATTAGCCCAACTGTATATAAATAATG |
| Q11A-1as | CATTATTTATATACAGTTGGGCTAATTGGAGAGG |
| Q12A-2s | CCTCTCCAATTACAGGCCCTGTATATAAATAATG |
| Q12A-1as | CATTATTTATATACAGGGCCTGTAATTGGAGAGG |
| L13A-2s | CCTCTCCAATTACAGCAAGCTTATATAAATAATG |
| L13A-1as | CATTATTTATATAAGCTTGCTGTAATTGGAGAGG |
| Y14A-2s | CCTCTCCAATTACAGCAACTGGCCATAAATAATG |
| Y14A-1as | CATTATTTATGGCCAGTTGCTGTAATTGGAGAGG |
| H25A-2s | CCTAATCCAGGAGCCGGAGATATTATTGCTGTCAGATTTAC |
| H25A-1as | GTAAATCTGACAGCAATAATATCTCCGGCTCCTGGATTAGG |
| G26A-2s | CCTAATCCAGGACATGCCGATATTATTGCTGTCAGATTTAC |
| G26A-1as | GTAAATCTGACAGCAATAATATCGGCATGTCCTGGATTAGG |
| D27A-2s | CCTAATCCAGGACATGGAGCCATTATTGCTGTCAGATTTAC |
| D27A-1as | GTAAATCTGACAGCAATAATGGCTCCATGTCCTGGATTAGG |
| I28A-2s | CCTAATCCAGGACATGGAGATGCCATTGCTGTCAGATTTAC |
| I28A-1as | GTAAATCTGACAGCAATGGCATCTCCATGTCCTGGATTAGG |
| I29A-2s | CCTAATCCAGGACATGGAGATATTGCCGCTGTCAGATTTAC |
| I29A-1as | GTAAATCTGACAGCGGCAATATCTCCATGTCCTGGATTAGG |
| Gag mut-1s | GAAGCCAGAACTCACATGAGTGGTG |
| Gag mut-2as | CATACGAGTGACTTGGTCC |
| **Cloning of src proviral Gag mutants** | |
| Src(+) | AAATTAAGCTGAGGAGAATAATCCCTAGGGACCTTACCTTACT  GAGGAAGGATGGGCAGCAGCAAGAGCAAGCCCAAGATGGC |
| Src(-) | **TCGA**GCCATCTTGGGCTTGCTCTTGCTGCTGCCCATCCTTCCTCA  GTAAGGTAAGGTCCCTAGGGATTATTCTCCTCAGCTTA |
| **Cloning of N-terminal deletion Gag mutants** | |
| E4A∆4 | TGGCTCGAGCGAATCCTCTCCAATTACAG |
| E4A∆7 | TGGCTCGAGCGCTCCAATTACAGCAACTG |
| E4A∆9 | TGGCTCGAGCGTTACAGCAACTGTATATAAA |
| E4A∆11 | TGGCTCGAGCGCAACTGTATATAAATAATGGC |
| AS-Primer | CCTAGGTTGAATGCAGTTTGT |

Underlined characters indicate a SwaI single-stranded overhang. Bold characters indicate an XhoI single-stranded overhang.
